# The Komodo dragon (*Varanus komodoensis*) genome and identification of innate immunity genes and clusters

**DOI:** 10.1186/s12864-019-6029-y

**Published:** 2019-08-30

**Authors:** Monique L. van Hoek, M. Dennis Prickett, Robert E. Settlage, Lin Kang, Pawel Michalak, Kent A. Vliet, Barney M. Bishop

**Affiliations:** 1 School of Systems Biology, George Mason University, Manassas, VA 20110 USA; 2 Dipartimento di Scienze della Vita-Edif. C11, Università di Trieste, Via Licio Giorgieri 1, 34127 Trieste, Italy; 3 Advanced Research Computing, Virginia Polytechnic Institute and State University, Blacksburg, VA 24061 USA; 4 Edward Via College of Osteopathic Medicine, Blacksburg, VA 24060 USA; 5 Center for One Health Research, Virginia-Maryland College of Veterinary Medicine, Blacksburg, VA 24060 USA; 6 Institute of Evolution, University of Haifa, 3498838 Haifa, Israel; 7 Department of Biology, University of Florida, Gainesville, Florida, FL 32611 USA; 8 Department of Chemistry, George Mason University, Manassas, VA 20110 USA

**Keywords:** Komodo dragon, *Varanus komodoensis*, Monitor lizard, Innate immunity, Antimicrobial peptide, Defensin, Cathelicidin, Gene cluster

## Abstract

**Background:**

We report the sequencing, assembly and analysis of the genome of the Komodo dragon (*Varanus komodoensis*), the largest extant lizard, with a focus on antimicrobial host-defense peptides. The Komodo dragon diet includes carrion, and a complex milieu of bacteria, including potentially pathogenic strains, has been detected in the saliva of wild dragons. They appear to be unaffected, suggesting that dragons have robust defenses against infection. While little information is available regarding the molecular biology of reptile immunity, it is believed that innate immunity, which employs antimicrobial host-defense peptides including defensins and cathelicidins, plays a more prominent role in reptile immunity than it does in mammals. .

**Results:**

High molecular weight genomic DNA was extracted from Komodo dragon blood cells. Subsequent sequencing and assembly of the genome from the collected DNA yielded a genome size of 1.6 Gb with 45x coverage, and the identification of 17,213 predicted genes. Through further analyses of the genome, we identified genes and gene-clusters corresponding to antimicrobial host-defense peptide genes. Multiple β-defensin-related gene clusters were identified, as well as a cluster of potential Komodo dragon ovodefensin genes located in close proximity to a cluster of Komodo dragon β-defensin genes. In addition to these defensins, multiple cathelicidin-like genes were also identified in the genome. Overall, 66 β-defensin genes, six ovodefensin genes and three cathelicidin genes were identified in the Komodo dragon genome.

**Conclusions:**

Genes with important roles in host-defense and innate immunity were identified in this newly sequenced Komodo dragon genome, suggesting that these organisms have a robust innate immune system. Specifically, multiple Komodo antimicrobial peptide genes were identified. Importantly, many of the antimicrobial peptide genes were found in gene clusters. We found that these innate immunity genes are conserved among reptiles, and the organization is similar to that seen in other avian and reptilian species. Having the genome of this important squamate will allow researchers to learn more about reptilian gene families and will be a valuable resource for researchers studying the evolution and biology of the endangered Komodo dragon.

**Electronic supplementary material:**

The online version of this article (10.1186/s12864-019-6029-y) contains supplementary material, which is available to authorized users.

## Background

**Fig. 1:**
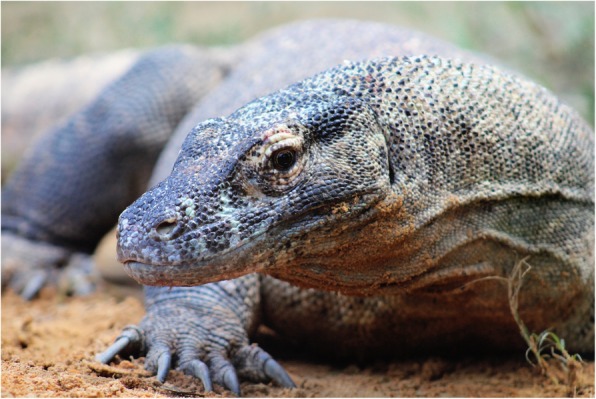
Komodo dragon (*Varanus komodoensis*). Tujah, a large male Komodo dragon residing at the St. Augustine Alligator Farm Zoological Park, and the source of the DNA used in the present study. Photograph courtesy of the St. Augustine Alligator Farm Zoological Park in St. Augustine, Florida

Komodo dragon (*Varanus komodoensis*) is the world’s largest extant lizard, weighing up to 75–100 kg and measuring up to three meters in length. This species of monitor lizard, indigenous to Komodo and nearby islands in southern Indonesia (Fig. 1), is a relic of very large varanids that once populated Indonesia and Australia, most of which, along with other megafauna, died out after the Pleistocene [1]. Komodo dragons are endangered and actively conserved in zoos around the world and in Komodo National Park, a UNESCO World Heritage site, due to their vulnerable status [2]. They are believed to have evolved from other varanids in Australia, first appearing approximately 4 million years ago [1].

On their native Indonesian islands, Komodo dragons are the dominant terrestrial predators, even though their diet is based mainly on carrion [3]. The saliva of wild dragons (as opposed to zoo-kept animals) has been found to contain as many as 58 species of bacteria, many of which are pathogenic [3–5], which may also contribute to their effectiveness as predators. The lizards themselves appear to be unaffected by these bacteria, despite biting each other in fights and having bleeding gums during feedings. Furthermore, their plasma has been shown to have potent antimicrobial properties [6]. Thus, we hypothesized that Komodo dragons would have robust innate immunity and this innate immunity may be partially mediated by antimicrobial peptides.

There are few studies regarding the reptilian immune response; however, as in mammals, reptiles have both an innate and adaptive immune response with cell mediated and humoral components. The reptile immune response is primarily dependent on an efficient innate immune response as the adaptive immune response does not consistently demonstrate evidence of a memory response [7].

Innate immunity, which includes chemokines and cytokines, provides the first line of defense against infection in higher vertebrates and is partially mediated by antimicrobial host-defense peptides [8, 9]. Antimicrobial host-defense peptides play complex roles in host defense against infection, with peptides exhibiting a range of pathogen-directed antimicrobial effects as well as host-directed immunomodulatory, chemotactic, inflammomodulatory and wound healing properties [8, 9]. The role and prevalence of antimicrobial peptides in the innate immune response of reptiles is only now being understood [10–15]. The plasma and cell extracts of crocodiles, alligators and Komodo dragons have been shown by several groups to have antimicrobial properties [6, 10, 16–20]. Recently, our group has made significant technical advances in developing a method for the identification and characterization of native antimicrobial peptides (BioProspector process), which we employed in the discovery of novel, non-canonical, active antimicrobial peptides in alligator plasma [21–23] and Komodo dragon plasma [24, 25].

The major classes of antimicrobial host-defense peptides in vertebrates include defensins and cathelicidins [8, 9]. These peptides are produced as part of the host-defense innate immune response by cells throughout the body, including epithelium, endothelium and white blood cells. Like most cationic antimicrobial host-defense peptides, defensins and cathelicidins tend to be relatively small peptides (< 100 amino acids in length) that simultaneously exhibit cationic and amphipathic qualities. They are generally membrane-active peptides that can disrupt bacterial membrane integrity as part of their antimicrobial mechanism. The cationic and amphipathic properties of these peptides contribute to their ability to preferentially target and disrupt bacterial membranes, which tend to be rich in anionic lipids, rather than host cell membranes, whose outer surfaces tend to be predominantly neutral in nature.

The family of vertebrate defensin peptides includes alpha-, beta-, theta- and ovo-defensin subclasses, with alpha- and theta-defensins being unique to mammals and ovodefensins to birds and reptiles [26, 27]. Peptides in each subclass exhibit compact three-dimensional conformations stabilized by characteristic conserved patterns of cysteine residues and associated disulfide bond networks. The disulfide bond networks in each defensin subclass are critical to their ability to adopt well-defined structures, which are essential to their antimicrobial and host-directed properties.

Cathelicidins are another major class of host-defense antimicrobial peptides and are unique to vertebrates [28]. The functional cathelicidin peptides exhibit diverse sequences and structures. However, they are distinguished by the presence of conserved N-terminal pre-pro-cathelin domains in the cathelicidin precursor proteins [29]. Cathelicidins are often packaged in azurotrophic granules in neutrophils and have been identified in chicken heterophils (avian white blood cells) [30]. The detailed characteristics of each peptide subclass are described in the relevant sections below.

Advances in genomic techniques and the availability of sequenced genomes have rapidly expanded our understanding of the presence of innate immunity genes across different classes. The anole lizard has been found to have genes for most of the major classes of antimicrobial peptides that are produced by mammals and other vertebrates, including β-defensins and cathelicidins [13]. As in the case of birds, genes for α-defensin peptides have not been reported to date in reptiles; this class of antimicrobial peptides appears to be restricted to mammals [13]. However, the status of antimicrobial peptide genes in the Komodo dragon has not been determined, due to the lack of a published Komodo dragon genome. Their tolerance to regular exposure to potentially pathogenic bacteria in their saliva and apparent resistance to bacterial infection suggests that Komodo dragon’s evolutionary adaptations may extend to their innate immunity and the host-defense peptides that they employ.

As part of our effort to extend our earlier study of Komodo dragon cationic antimicrobial peptides [24], genomic DNA and RNA were obtained from Komodo dragon blood samples and sequenced in order to provide a Komodo dragon-specific DNA sequence database to facilitate de novo peptide sequencing [24].

Here, we report the sequencing, assembly, and analysis of the Komodo dragon genome. This work will also provide evidence of the robust innate immunity of these lizards and will be a valuable resource for researchers studying the evolution and the biology of the endangered Komodo dragon. The analysis reported here is focused on genes associated with innate immunity and host-defense peptides. However, further investigation of the Komodo dragon genome may have broader impact on our understanding of the biology and evolution of reptiles.

## Results and discussion

### Cell types in Komodo dragon blood

A sample of blood was obtained from a Komodo dragon named Tujah at the Saint Augustine Alligator Farm Zoological Park in accordance with required safety and regulatory procedures, and with appropriate approvals. At the time of collection, we were interested in collecting both genomic DNA for sequencing as well as mRNA to generate a cDNA library to facilitate our proteomic studies. In birds, the heterophils (white blood cells) are known to express multiple antimicrobial peptides [30]. Antimicrobial peptides identified from chicken heterophils exhibit significant antimicrobial [31, 32] and host-directed immunomodulatory activities [29]. Accordingly, after obtaining an initial sample of fresh Komodo dragon blood, we allowed the white blood cells to settle out of the blood and collected them because they were likely to be involved with antimicrobial peptide expression. The collected Komodo dragon white blood cells were then divided evenly, with half being processed for the isolation of genomic DNA in preparation for sequencing and library generation, and the other half reserved for mRNA extraction for our proteomic studies.

**Fig. 2:**
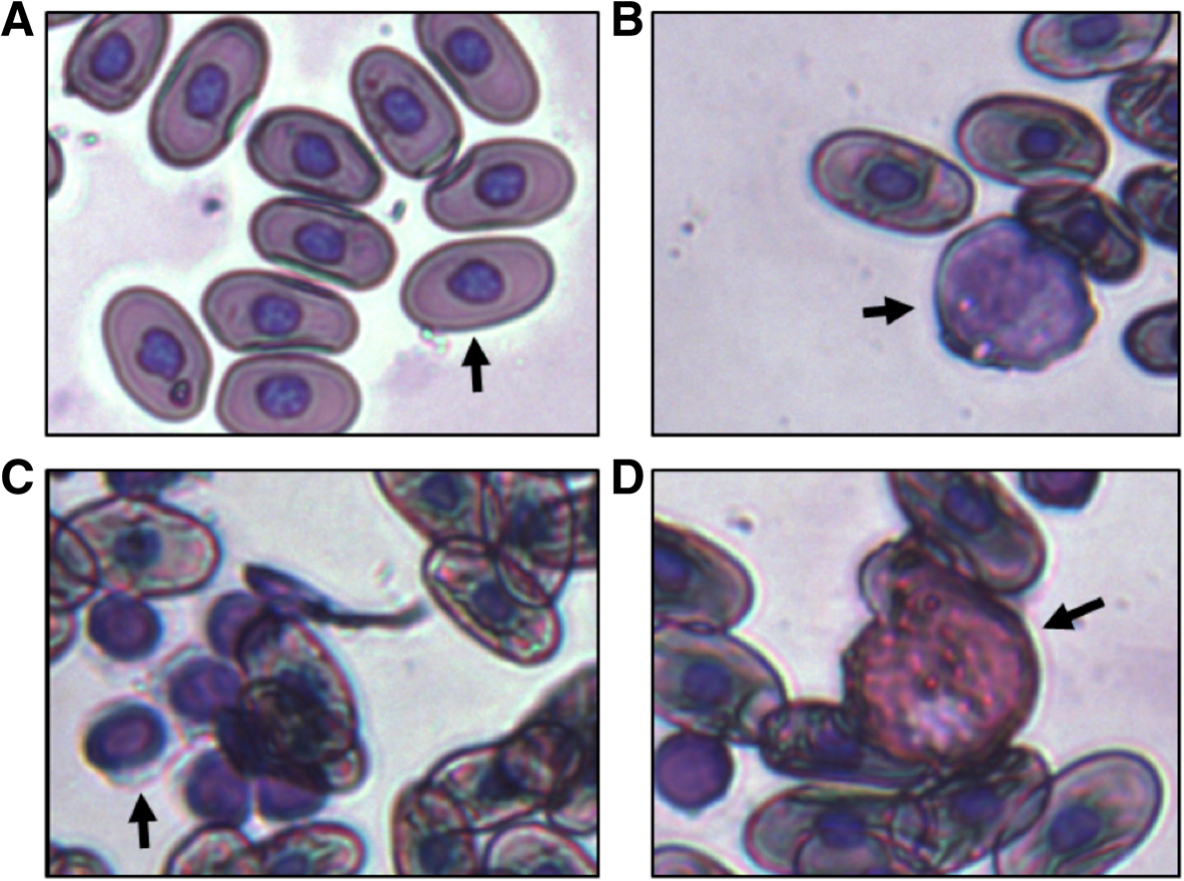
Komodo dragon red blood cells and immune cells. Blood cells from Komodo dragon were visualized by Wright stain and imaged at 40x. Cell types are identified as: A. nucleated red blood cell, B. monocyte, C. lymphocyte, and D. heterophil

We then performed smears and identified the various cell types that we observed. Immune cell identification in Komodo dragon blood is challenging due to limited published literature for reference. The various cell types that were observed in Wright-stained blood smears are shown in Fig. 2. We identified these cells based on similarity to the immune cells we had previously identified in the American alligator blood [12]. Of interest were the large and elongated nucleated red blood cells of this reptile. In addition, we were able to identify heterophils (similar to granulocytes), a probable source of cathelicidin peptides, as well as monocyte and lymphocyte cells.

A second sample of Komodo dragon blood was later collected and processed for genomic DNA extraction by Dovetail Genomics for additional sequencing. The researchers at Dovetail Genomics did not separate white blood cells, and instead extracted DNA from cells pelleted directly from whole blood.

### Assembly and annotation of the Komodo dragon genome

Previous analyses of Komodo dragon erythrocytes using flow cytometry estimated the genome to be approximately 1.93 Gb in size [33]. Using deep Illumina sequencing and Dovetail approaches, we obtained a draft genome assembly that was 1.60 Gb large, similar to the genome size of *A. carolinensis* lizard genome which is 1.78 Gb [34]. The draft assembly contains 67,605 scaffolds with N50 of 23.2 Mb (Table 1). A total of 17,213 genes were predicted, and 16,757 (97.35%) of them were annotated. Completeness estimates with CEGMA [35] were 56% (‘complete’) and 94% (‘partial’). The estimated percentage of repeats in the genome is 35.05% with the majority being LINEs (38.4%) and SINEs (5.56%) (Additional file 1: Fig. S1 & Additional file 2: Table S1). Genomic data will be available at NCBI with raw sequencing reads deposited in the Sequence Read Archive (#SRP161190), and the genome assembly at DDBJ/ENA/GenBank under the accession #VEXN00000000. The assembly version described in this paper is VEXN01000000.

**Table 1:** Genome assembly attributes

Assembly Attributes	
Total Size	1599,705,180 bp
No. of Scaffold	67,605
No. of Scaffold > 5 K	2608
Scaffold N50	23,221,552 bp
GC Content	44.30%
No. of Predicted Gene	17,213
No. (Percentage) of Annotated Gene	16,757 (97.35%)

### Identification of potential innate immunity and antimicrobial peptide genes

Innate immunity in reptiles is a critical aspect of their evolutionary success, but it remains poorly understood in these animals. Innate immunity is defined as those aspects of immunity that are not antibodies and not T-cells. Innate immune responses to invading pathogens can include the expression of cytokines; the activation and recruitment of macrophages, leukocytes and other white blood cells; and the expression of antimicrobial peptides such as defensins and cathelicidins [13, 15].

We have taken a genomics-based approach [36] to identifying innate immunity genes in the Komodo dragon genome in this work. We have sequenced the Komodo genome and examined it for genes and clusters of important innate immunity antimicrobial peptide genes (β-defensins, ovodefensins and cathelicidins), which are likely involved in expressions of innate immunity in this giant lizard.

#### β-Defensin and related genes in Komodo genome

Defensins are one example of disulfide-stabilized antimicrobial peptides, with β-defensins being a uniquely vertebrate family of disulfide-stabilized, cationic antimicrobial peptides involved in the resistance to microbial colonization at epithelial surfaces [37–39]. The β-defensin peptides are defined by a characteristic six-cysteine motif with conserved cysteine residue spacing (C–X6–C–X (3–5)–C–X (8–10)–C–X6–CC) [40] and associated disulfide bonding pattern (Cys1-Cys5, Cys2-Cys4 and Cys3-Cys6); however, variations in the number of and spacing between cysteine residues has been observed. As with other cationic antimicrobial peptides, β-defensins typically exhibit a net positive (cationic, basic) charge.

One of the first extensive reports of an in vivo role for β-defensin peptide expression in reptiles is the inducible expression of β-defensins in wounded anole lizards (*Anolis carolinensis*) [10, 11, 14, 41–43]. Reptile neutrophils appear to have granules that contain both cathelicidin-like peptides as well as β-defensin peptides. β-defensin-like peptides are also found in reptile eggs [26]. It is well-known that some species of lizard can lose their tails as a method of predator escape, and that these tails then regenerate from the wound site without inflammation or infection. β-defensin peptides are expressed both within the azurophilic granulocytes in the wound-bed as well as in the associated epithelium [41, 43] and are observed in phagosomes containing degraded bacteria. There is a distinct lack of inflammation in the wound, which is associated with regeneration, and two β-defensins in particular are expressed at high levels in the healing tissues [10, 42] Overall, there appears to be a significant role for the β-defensins in the wound healing and regeneration in the anole lizard [44].

β-defensin genes have been generally observed to reside in clusters within the genomes of vertebrates [45, 46]. In humans, as many as 33 β-defensin genes were identified in five clusters [47, 48]. Recently, analyses of the genomes of several avian species including duck, zebra finch and chicken revealed that the genome of each species contained a β-defensin cluster [49–52]. A β-defensin-like gene cluster has recently been identified in the anole lizard (Prickett, M.D., *unpublished work in progress*), which is closely related to the Komodo dragon [13]. Interestingly, the cathepsin B gene (CTSB) has been identified as a strong marker for β-defensin clusters in humans, mice, and chickens [51]. Thus, we examined the Komodo genome for the cathepsin B gene (CTSB) as a potential marker to aid in the identification of the β-defensin cluster(s) therein.

Through these analyses, we identified a total of 66 potential β-defensin genes in the Komodo dragon genome, of which 18 are thought to be Komodo dragon-specific β-defensin genes (Table 2). The β-defensin genes identified from the Komodo dragon genome exhibit variations in cysteine spacing, gene size, the number of cysteine residues that comprise the β-defensin domain, as well as the number of β-defensin domains. With respect to the conserved cysteine residue spacing, especially at the end (C–X6–C–X (3–5)–C–X (8–10)–C–X6–CC), we found considerable variability in our analysis of the β-defensin genes in the Komodo dragon genome, in that five Komodo dragon β-defensin genes have seven resides between the last cysteines, 16 have six residues between the last cysteines, 42 have five residues between the last cysteines, and three Komodo dragon β-defensin genes exhibit more complex cysteine-residue spacing patterns **(**Table 2**).**

**Table 2:** Identified Komodo dragon Defensin genes grouped based on scaffold locations of gene clusters

Name	Closest Anole Ortholog	Closest Snake Ortholog	Paralogs	Sequences
Scaffold 210	This scaffold contains the cluster of six β-ovodefensin genes, one intercluster β defensin, and 15 β-defensin genes that are similar to anole lizard β-defensins. The cluster begins with the CTSB gene. These genes include large β defensins.			
VkBDic1_VARCO	LzBD81	SnBDic1,2		GLAKRKPRSRRECYSLDGSCYLGRCPSVLKKYGWCGTLKRCCIR
VkBD1_VARKO	LzBD1	SnBD1,		AMGDLYDSLECHNHHGHCRRICFHNERSIGTCTNRRQLCCK
VkBD2_VARKO	LzBD2	SnBD2,	VkBD3,4,5,6	GKGDFVTLGCLFRGGACKTDTCRDNEVQIGNCSNTKQLCCKKTR
VkBD3_VARKO	LzBD3		VkBD2,4,5,6	GALQDKKGSCDLALHNCRMGFCSQEEIPTGEFCFEPVILCCKHLPEKSKSPERLEGVASFADVLRNLL
VkBD4_VARKO	LzBD4	SnBD3,	VkBD2,3,5,6	GGGQREARFVSHCLRRGGICRYDDCSEGEEQIGTCYHHTMICCRDEVM
VkBD5_VARKO	LzBD4	SnBD3,4,	VkBD2,3,4,6	GASLNLSARICWQRGGRCRSSPCYYDEAQIGTCYHARLKCCRQTE
VkBD6_VARKO	LzBD5		VkBD2,3,4,5	GHSNEIDTQQCLKDKGTCHPTICPTQKMSKGTCYSGAQLCCVGESVHMCVRPFFPSCCFSRLSCSRSGGL
VkBD7_VARKO	LzBD6,82	SnBD5,6,	VkBD8	GQPHICKRMGGYCQITSKPCPYGFLPTTCGIFQTCCAKKPPTTNDCEKLGGFCRVPLHEKCPSGQDLPANCGINARCCKPETA
VkBD8_VARKO	LzBD6,82	SnBD5,6,	VkBD7	GQVPACTKLGGHCVTPLTVTCPYGSLGANCGINAICCAR
VkBD9_VARKO				TIGTFLVIQAKLCRKAGGFCISRFAHCPSSEIILIRCRSAQKCCKQ
VkBD10_VARKO	LzBD11	SnBD7,		EGCPVEAMLLTCTSLGGFCMLEPNQTCPSGLILDVPCHFKRRCCSKKEV
VkBD11_VARKO				GQSLSCRELGGLCVALADDDCDSEEILPADCGRLTLCCKG
VkBD12_VARKO	LzBD12–17	SnBD8 (python only)	VkBD13,14	EQALRRVPPVQIRSIRRYCNGRDGYCLDRKFFCESGFVYKEEYNDCMFQNKNKCCVIQKSK
VkBD13_VARKO	LzBD12–17	SnBD8 (python only)	VkBD12,14	DPSLPSKKAATSRFTTFACNGNFGYCLPRNWRCFSGLVYKEEFNDCPLPDIFKCCL
VkBD14_VARKO	LzBD12–17	SnBD8 (python only)	VkBD12,13	NKGLPTTTRVGKIPCLGPWGHCFFRKYRCASGFVMKERFNNCPNTRTLKCCVL
VkBD15_VARKO				GFLHVLARHCQKLGGTCKPHKAQCKVRIQILVPCGPGRKCCL
Scaffold 286	We identified 40 β defensins genes, including multiple large defensins (with some peptides containing eight instead of typical six cysteine residues) and peptides with multiple domains.			
VkBD16_VARKO			VkBD17, 18	GGGLQDGEDPPGPRASCNSGRGYCLRCGAACPSGQIYVYNDCANPCSNKCCVRR
VkBD17_VARKO			VkBD16,18	GQSSYWGHLSCNSGRGYCLPCHLRCPYGYYYYNDCPGQCRYKCCVRR
VkBD18_VARKO			VkBD16,17	APDRVPHEQAPPCNHGLGYCHPCSLPCTSGKYYYYNDCDKPCANRCCTKQ
VkBD19_VARKO				GTELMEEEYLARAILCHSGQGVCRPRDLQCPSGLTYIYNDCPKTELYKCCAK
VkBD20_VARKO				EQNRLARSKCVCRKFCYPNEYPMGMCAIILVPMCCTFDPQGGS
VkBD21_VARKO			VkBD22	VSDKSSSCRSAGGKCYLILCPRGTGRIGKCSFTHVCCK
VkBD22_VARKO			VkBD21	GRTEVHDVRMCKLYGGECFLLVCPPGRAFIGKCSRLNVCCRG
VkBD23_VARKO	LzBD26	SnBD9, 10 (Colubroidea only)		GLAPDTPHDRSSCELNSGFCYSSNCPRCFGQYGFCDDKHPSCCVRDNSIPGCEVVTTPTYDKSSKTTAVPTTSETAPPQTAPPQTAPPQTAPPQTAPDESDKKC
VkBD24_VARKO	LzBD27			GYGLVPAGLSDTIKCRTTPRSFCKAVVCPPTFEPTGTCFGGSMHCCSK
VkBD25_VARKO				GEFANISNYYQCRHAGAMCAIFKCPAPFNSIGKCGIFKPCCI
VkBD26_VARKO				GSTEIIGEAKCAEYTGTCRLFECPISSMVENIGYCREDYVCCIR
VkBD27_VARKO	LzBD31			DGGTCRQLKGFCSTRRCPDKTFEVLGRCTPRKACCQKKANA
VkBD28_VARKO	LzBD32,33	SnBD13,14		GNAQAAEPDTLQCVRAGGSCNFGECRPPSVASGTCKGETLNCCKW
VkBD29_VARKO	LzBD34,35, 37,73		VkBD30,31	GHSVSNEAECKREGGFCIIAIGNPCRFPYGFIGKCSWWKFCCK
VkBD30_VARKO	LzBD34,35, 37,73		VkBD29,31	GSARWVKNEDECKEDGGTCLAIVIGGCGPLLQIGICGLGRNCCK
VkBD31_VARKO	LzBD34,35, 37,73		VkBD29,30	GCSISDRKECREGGGFCLPFLPNMCQVFSIIIGKCSRWSYCCKW
VkBD32_VARKO				ASTINTSQQCKNAGGHCIWSSCGFPLCPVGRCGYWSLCCKA
VkBD33_VARKO				GTPASPVRGPLQCVKQGGFCMSGNCRFPLRKLGTCHRFKACCIR
VkBD34_VARKO	LzBD39	SnBD15		GIYTTLDLEMLSCLSAKDAYCKTGHCLEYSVSLGKCNSYLSCCRSMINNLWRCAIAKGYCSMWTCPPPLIKNGRCSRDSPCCVQ
VkBD35_VARKO	LzBD41,43	SnBD17 (Colubroidea only)	VkBD36	GLAMRTERIDSAEECAKIQGFCTSEPECNTVYPIQGTCGEGTLCCLE
VkBD36_VARKO	LzBD41,43	SnBD17 (Colubroidea only)	VkBD35	TGYTCVLEEILTKEDCELIGATCIDGEDCNPPFPSQGTCGEGTVCCIP
VkBD37_VARKO	LzBD50			GLTTYVTSPEECESIKGFCTPRLCQENFRTLGECSAGIPCCTR
VkBD38_VARKO		SnBD17 (python only)		GFTRDIWDRPTCRSSQGFCWLITCPWPFAKHIGECIWPILRCCA
VkBD39_VARKO	LzBD52,53,55 (2 defensin domains)		VkBD43	DLAADCRLREGRCTRTSCSKTDIYLGECAKEIQCCKRDPALHCERQGGLCTQASCTGSDINLGPCGRGLKCCKKDPVAHCEAQGGKCVQNSCPTSDVFLGRCGNGFQCCKT
VkBD40_VARKO	LzBD52–55			GQPQKYIEKKECMDFGAQCTKAHCGEDYIFYGFCRTGVACCKR
VkBD41_VARKO	LzBD57	SnBD22 (pseudo-gene)		GVALPFQDDSVCIERGGRCMHLPCHPLRRIGRCLLNTYCCH
VkBD42_VARKO	LzBD58			GSTQFIKLAHKCLDAGGRCQKQACDFRKSIGRCNDQELCCKR
VkBD43_VARKO	LzBD52,53,55 (2 defensin domains)		VkBD39	DPVASCASQGGKCTPDICPSNFVLVGQCDQKLLCCKRDPVAHCKSQGGRCMQSRCASNNEYLGDCGPAVWCCKM
VkBD44_VARKO				GFAHMGINFREQCQWAEGKCQLYHCPAGWKKIGKCSKVVPCCSDK
VkBD45_VARKO				GSNPKQCEQKRAQCMIVCPRTHKKVGHCGRSLSCCAQR
VkBD46_VARKO				GYTSAVARCRRRGGKCHFGRCPQGKNRIGLCFLGTPCCTR
VkBD47_VARKO	LzBD63			GFTTEIRRARTCIEANGKCRLATCLHDPWERIGKCGDKRFCCKK
VkBD48_VARKO			VkBD50,52, 53,54	GHTFGFLRCRRNGGHCFPRGCPVGWKPRGRCIGRFTCCVRQGKRPAMEKARQ
VkBD49_VARKO				GHTFGVGECRDQFGSCKFAVCRNGWRRVGWCFLAVPCCRR
VkBD50_VARKO			VkBD48,52, 53,54	GTGQTPGALRCQRNGGLCFPWGCPPGWRPRGRCFGRFTCCIR
VkBD51_VARKO				GRTSGLVACQRKRGICLVGRCRPGWRQVGWCSRGVSCCKW
VkBD52_VARKO			VkBD48,50, 53,54	GQKPVILRCLRNGGRCFSWRCPSGWMLRGPCLRPFICCVR
VkBD53_VARKO			VkBD48,50, 52,54	GRTFGPPRCRRIGGFCIPRRCPSGWRQRGPCLGPFTCCKR
VkBD54_VARKO			VkBD48,50, 52,53	GTARTFGPARCRRIGGLCIPWGCPSGWRRRGRCFGRFTCCKR
VkBD55_VARKO				GGSSDGQPVSAPSSARLMRRCRSFYSPCKTCPVLLKELSCQVVHHPCCPPLKLLHL
Scaffold 7	We identified seven β-defensins on the edge of a large scaffold. This cluster ends with the gene XHO1			
VkBD56_VARKO			VkBD58,60	GCLGVYEKGRRQCCTRNGHCYFLFCKKGTVKIGTCNFFSRCCSR
VkBD57_VARKO				GLAGENELDQRGCRRRCLVRHCTRRGRFYVCRRYYICCGR
VkBD58_VARKO			VkBD56,60	ECLGVYVKGRRQCRTQNGHCYFFYCKKDTYKIGICNFFTICCSR
VkBD59_VARKO				VSSPEECGRHGGLRLAGPRFSCWSGLLTGHCDAKHRCCKR
VkBD60_VARKO			VkBD56,58	ECHGMYVNGKRQCHTQNGHCYFLYCKKDTYKIGTCNFFSRCCSR
VkBD61_VARKO	LzBD77			GFSWSNDATLRCRYTGGYCDSLICRWPLRNAGFRCKNNRPCCKR
VkBD62_VARKO	LzBD78			GSARTPRSDLECQVHKGMCFPHGCPAQWSRIGSCSVRKHRCCR
Scaffold 45	We identified three β-defensin genes and TRAM2 on the edge of a large scaffold. The gene VKBD80 may be a duplication. TRAM2represents the end of this cluster.			
VkBD79_VARKO				PGPYPCNWVCHTSPGVQSVCSAMPVRLKLLLGLASVCISVSTLCCFRQH
VkBD80a_VARKO	LzBD80	SnBD40,		GRCRRLKGVCRHTLCHPVEVYVGRCNNGMGNCCVDDAEDIRKHIK
VkBD80b_VARKO	LzBD80	SnBD40,		GRRRRLKGVCRHTLCHPVEVYVGRCNNGMGNCCVDDAEDIRKHIK

Reptile β-defensin orthologs are also indicated (where they exist) as well as the β-defensin peptide sequences encoded by each gene. Overall, 66 β-defensin genes were identified, of which 4 encode multiple defensin domains (VkBD7, VkBD34, VkBD39, and VkBD43)

**Fig. 3:**
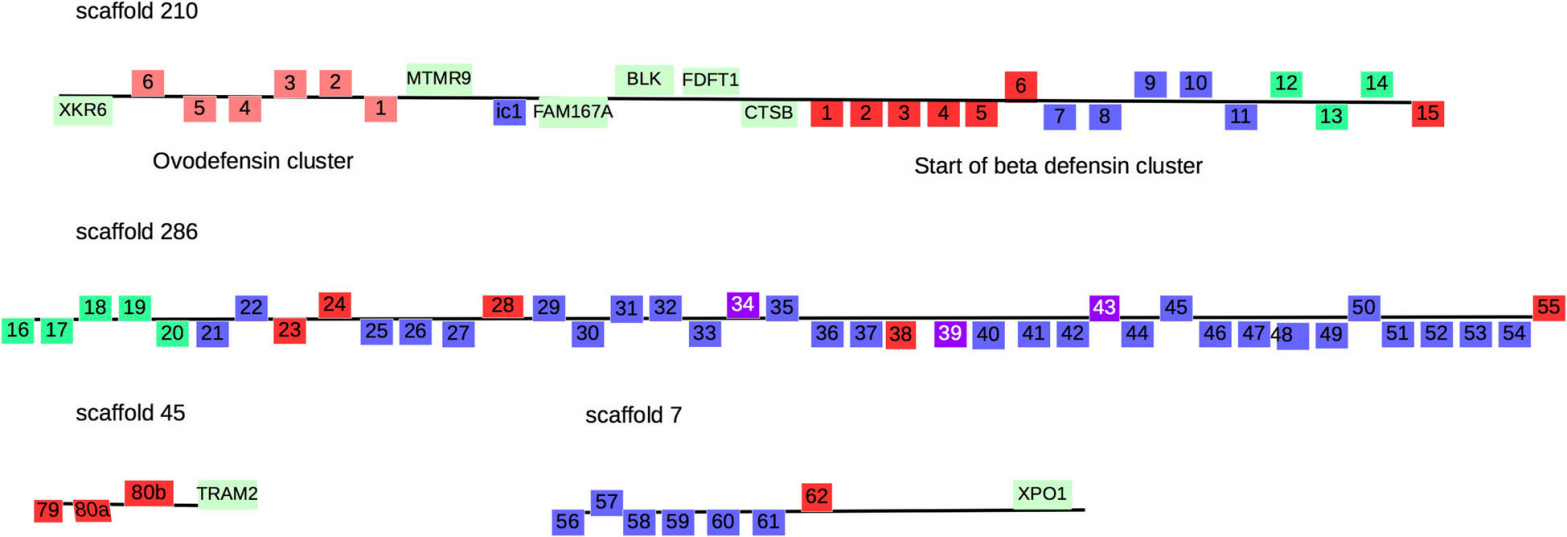
β-defensin gene family clusters. Scaffold locations of the identified Komodo dragon defensin and ovodefensin genes, highlighting the defensin and ovodefensin clusters in the Komodo dragon genome

As with birds and other reptiles, the majority of Komodo dragon defensin genes appear to reside in two separate clusters within the same syntenic block (Fig. 3). One cluster is a β-ovodefensin cluster flanked on one end by the gene for XK, Kell blood group complex subunit-related family, member 6 (XKR6) and on the other end by the gene for Myotubularin related protein 9 (MTMR9). The intercluster region of circa 400,000 bp includes the genes for Family with sequence similarity 167, member A (FAM167A); BLK proto-oncogene, Src family tyrosine kinase (BLK); Farnesyl-diphosphate farnesyl transferase 1 (FDFT1); and CTSB (cathepsin B), which is a flanking gene for the β-defensin cluster (Fig. 3). In birds, turtles, and crocodilians, the other end of the β-defensin cluster is followed by the gene for Translocation associated membrane protein 2 (TRAM2). As is the case with all of the other squamate (lizards and snakes) genomes surveyed, the flanking gene for the end of the β-defensin cluster cannot be definitively determined at present as there are no squamate genomes with intact clusters available.

The end of the cluster could either be flanked by XPO1 or TRAM2 or neither. Two of the three genes found on scaffold 45 with TRAM2 (VkBD80a, VkBD80b) are nearly identical and potentially the result of an assembly artifact. The genes are orthologs for the final gene in the avian, turtle, and crocodilian β-defensin clusters. The anole ortholog for this gene is isolated and is not associated with TRAM2, XPO1, nor any other β-defensins, and there are no β-defensins found in the proximity of anole TRAM2. Two of the seven genes associated with XPO1 have orthologs with one of the five anole genes associated with XPO1 but it cannot be determined in either species if these are part of the rest of the β-defensin cluster or part of an additional cluster. The snake orthologs are associated with TRAM2 but are not part of the cluster.

### Structural diversity

Diversity can be seen in variations in structure of the β-defensin domain. Typically, a β-defensin consists of 2–3 exons: a signal peptide, an exon with the propiece and β-defensin domain with six cysteines, and in some cases, a short third exon. Variations in the number of β-defensin domains, exon size, exon number, atypical spacing of cysteines, and/or the number of cysteines in the β-defensin domain can be found in all reptilian species surveyed (unpublished). There are three β-defensins with two defensin domains (VkBD7, VkBD34, and VkBD43) and one with three defensin domains (VkBD39). The Komodo dragon β-defensin genes VkBD12, VkBD13, and VkBD14 and their orthologs in anoles have atypically large exons. The group of β-defensins between VkBD16 and VkBD21 also have atypically large exons. Atypical spacing between cysteine residues is found in three β-defensins, VkBD20 (1–3–9-7), VkBD57 (3–4–8-5), and VkBD79 (3–10–16-6). There are four β-defensins with additional cysteine residues in the β-defensin domain: VkBD6 with 10 cysteine residues, and a group of three β-defensins, VkBD16, VkBD17, and VkBD18, with eight cysteine residues.

The two β-defensin domains of VkBD7 are homologous to the one β-defensin domain of VkBD8 with orthologs in other species of Squamata. In the anole lizard *A. carolinensis* there are two orthologs, LzBD6 with one β-defensin domain and the non-cluster LzBD82 with two β-defensin domains. The orthologs in snakes (SnBD5 and SnBD6) have one β-defensin domain. VkBD34 is an ortholog of LzBD39 in anoles and SnBD15 in snakes. VkBD39 and VkBD43 consist of three and two homologous β-defensin domains respectively, which are homologous to the third exons of LzBD52, LzBD53, and LzBD55, all of which have two non-homologous β-defensin domains. VkBD40 with one β-defensin domain is homologous to the second exons of LzBD52, LzBD53, LzBD54 (with one defensin domain), and LzBD55.

An increase in the number of cysteines in the β-defensin domain results in the possibly of forming additional disulfide bridges. Examples of this variation can be found in the psittacine β-defensin, Psittaciforme AvBD12 [52]. The β-defensin domain of VkBD6 appears to consist of 10 cysteines, four of which are part of an extension after a typical β-defensin domain with an additional paired cysteine (C-X6-C-X4-C-X9-C-X6-CC-X7-C-X7-CC-X5-C). The group of Komodo β-defensins VkBD16, VkBD17, and VkBD18, in addition to having an atypical cysteine spacing, also have eight cysteines within a typical number of residues. The β-defensin following this group, VkBD19, is a paralog of these three genes; however, the β-defensin domain contains the more typical six cysteine residues.

The gene structures of these Komodo β-defensin genes are subject to confirmation with supporting evidence. There are a number of atypical structure elements in anole lizards including additional non β-defensin domain exons or larger exons.

Analyses of the peptide sequences encoded by the newly identified Komodo dragon β-defensin genes revealed that the majority (53 out of 66) of them are predicted to have a net positive charge at physiological conditions, as is typical for this class of antimicrobial peptide (Table 3). However, it is notable that four peptides (VkBD10, VkBD28, VkBD30 and VkBD34) are predicted to be weakly cationic or neutral (+ 0.5–0) at pH 7, while nine peptides (VkBD3, VkBD4, VkBD11, VkBD19, VkBD23, VkBD26, VkBD35, VkBD36 and VkBD37) are predicted to be weakly to strongly anionic. These findings suggest while these peptides exhibit canonical β-defensin structural features and reside in β-defensin gene clusters, one or more of these genes may not encode for β-defensin-like peptides or canonical β-defensins, because β-defensins typically are cationic and their positive charge contributes towards their antimicrobial activity.

**Table 3:** Physical properties of identified β-defensin peptides

Defensin domain	Length	Molecular weight	Cys Residue Spacing	Charge	Isoelectric point
VkBD1_VARKO	41	4816.46	6–3–9-6	4.5	7.9826
VkBD2_VARKO	44	4770.49	6–4–9-6	4	8.5177
VkBD3_VARKO	68	7510.69	6–4–9-6	-1	5.635
VkBD4_VARKO	48	5466.13	6–4–9-6	−0.5	6.1876
VkBD5_VARKO	45	5112.8	6–4–9-6	4.5	8.5056
VkBD6_VARKO	70	7548.7	6–4–9-7-7 − 5	4.5	7.9825
VkBD7_VARKO	83	8922.43	6–6–8-5/6–6–7-5	6	8.2233
VkBD8_VARKO	39	3850.54	6–6–7-5	2.5	7.9391
VkBD9_VARKO	46	5087.15	6–6–9-5	7.5	9.7437
VkBD10_VARKO	49	5346.37	6–6–7-5	0.5	6.8341
VkBD11_VARKO	40	4130.68	6–7–9-5	-5	3.8346
VkBD12_VARKO	61	7362.5	6–6–12-7	6	9.0239
VkBD13_VARKO	56	6373.34	6–6–12-7	2	7.9229
VkBD14_VARKO	53	6051.22	6–6–12-7	9.5	10.3828
VkBD15_VARKO	42	4598.66	6–6–9-6	9.5	10.246
VkBD16_VARKO	54	5598.25	6–2–3-10-3-3	2	7.796
VkBD17_VARKO	47	5538.28	6–2–3-9-3-3	6	8.4828
VkBD18_VARKO	50	5606.31	6–2–3-10-3-3	2.5	7.512
VkBD19_VARKO	52	5871.76	7–6–10-7	−0.5	5.6992
VkBD20_VARKO	43	4861.77	1–3–9-7	2	7.9237
VkBD21_VARKO	38	4009.73	6–4–9-5	6.5	9.1233
VkBD22_VARKO	42	4645.55	6–4–9-5	4.5	8.5176
VkBD23_VARKO	104	11,026.09	6–4–9-6	−3	4.6839
VkBD24_VARKO	48	5012.86	7–4–9-6	3.5	8.2461
VkBD25_VARKO	42	4575.41	6–4–9-5	3.5	8.2423
VkBD26_VARKO	44	4914.62	6–4–11-5	−3	4.2446
VkBD27_VARKO	41	4535.29	6–4–10 − 5	7	9.6914
VkBD28_VARKO	45	4618.16	6–4–9-6	0	5.9979
VkBD29_VARKO	44	4958.8	6–7–9-5	4.5	8.5135
VkBD30_VARKO	44	4538.29	6–8–9-5	0	6.0306
VkBD31_VARKO	44	5009.92	6–7–10-5	3	8.1509
VkBD32_VARKO	41	4339.01	6–4–9-5	3.5	8.1572
VkBD33_VARKO	44	4782.74	6–4–9-5	8.5	10.5052
VkBD34_VARKO	84	9330.95	7–4–9-5/7–4–9-5	4.5	8.0587
VkBD35_VARKO	47	5058.7	6–5–9-5	-5	3.9254
VkBD36_VARKO	48	5026.65	6–5–9-5	−8	3.371
VkBD37_VARKO	43	4702.36	6–4–9-5	−1	4.7382
VkBD38_VARKO	44	5172.09	6–4–10-6	2.5	7.9416
VkBD39_VARKO	111	11,483.56	6–4–9-5/6–4–9-5/6–4–9-5	5	7.86
VkBD40_VARKO	43	4914.68	6–4–9-5	2.5	7.9327
VkBD41_VARKO	41	4641.48	6–4–8-5	3.5	7.9625
VkBD42_VARKO	42	4717.47	6–4–8-5	5.5	8.8017
VkBD43_VARKO	74	7874.1	6–4–9-5/6–4–9-5	2.5	7.65
VkBD44_VARKO	45	5073.96	6–4–9-5	5	8.5147
VkBD45_VARKO	38	4229.98	6–3–9-5	8	9.7441
VkBD46_VARKO	40	4388.17	6–4–9-5	9.5	10.8323
VkBD47_VARKO	44	5072.96	6–4–10-5	6.5	9.154
VkBD48_VARKO	52	5934.01	6–4–9-5	13	11.9504
VkBD49_VARKO	40	4566.32	6–4–9-5	5.5	8.814
VkBD50_VARKO	42	4627.4	6–4–9-5	7	10.4698
VkBD51_VARKO	40	4467.32	6–4–9-5	9	11.3419
VkBD52_VARKO	40	4623.65	6–4–9-5	8	11.1693
VkBD53_VARKO	40	4537.41	6–4–9-5	10	11.9738
VkBD54_VARKO	42	4760.65	6–4–9-5	11	12.1412
VkBD55_VARKO	56	6092.22	6–2–9-6	6.5	8.7963
VkBD56_VARKO	44	5031.94	3–4–8-5	8.5	9.2085
VkBD57_VARKO	40	4852.66	6–4–9-5	8.5	9.9206
VkBD58_VARKO	44	5254.16	6–7–8-5	6.5	8.8379
VkBD59_VARKO	40	4369.02	6–4–9-5	5.5	8.6986
VkBD60_VARKO	44	5232	6–4–10-5	6.5	8.6105
VkBD61_VARKO	44	5146.91	6–4–9-6	7	9.5242
VkBD62_VARKO	43	4829.59	3–10–16-6	7.5	9.1865
VkBD79_VARKO	49	5260.29	6–4–9-6	4	8.2526
VkBD80a_VARKO	45	5112.96	6–4–9-6	5.5	8.5212
VkBD80b_VARKO	45	5166	6–4–9-6	6.5	9.0493
VkBDic1_VARKO	44	5069.05	6–4–9-5	10	10.3026

Molecular weights, charges and isoelectric points were calculated based on predicted peptide sequences using the EMBOSS tool pepstats [53]

#### Identification of Komodo dragon ovodefensin genes

Ovodefensin genes have been found in multiple avian and reptile species [26], with expression found in egg white and other tissues. Ovodefensins including the chicken peptide gallin (*Gallus gallus* OvoDA1) have been shown to have antimicrobial activity against the Gram-negative *E. coli* and the Gram-positive *S. aureus*. Presumptive β-ovodefensins are found in a cluster in the same syntenic block as the β-defensin cluster in birds and reptiles. There have been 19 β-ovodefensins found in *A. carolinensis* (one with an eight cysteine β-defensin domain) and five in snakes (four with an eight cysteine β-defensin domain) (Prickett, M.D., *unpublished work in progress*). The Komodo dragon cluster consists of six β-ovodefensins (Tables 4 and 5). Two of these may be Komodo dragon specific; VkOVOD1, which is a pseudois an ortholog of SnOVOD1 in addition to the first β-ovodefensin in turtles and crocodilians. The defensin domains VkOVOD3, VkOVOD4, and VkOVOD6 consist of eight cysteines, orthologs of SnOVOD2, SnOVOD3, and SnOVOD5, respectively. VkOVOD4 and VkOVOD6 are orthologs of LzOVOD14.

**Table 4:** Ovodefensin peptides predicted in the Komodo dragon genome

Name	Closest Anole Ortholog	Closest Snake Ortholog	Sequences
Scaffold 210 Ovodefensins	This scaffold contains the cluster of six β-ovodefensin genes, one intercluster β defensin, and 15 β-defensin genes that are similar to anole lizard β-defensins. The cluster begins with the CTSB gene. These genes include large β defensins.		
VkOVOD1_VARKO	LzOVOD1		GM*PPHLCFLAMTLHLACSFCGMNFCAGAPRKLWCYCAAQRCCAL
VkOVOD2_VARKO		SnOVOD1	GRRRASDTQSCIRREGYCKPQCNETGPVPELNLGTCTNRQQKCCRPQEL
VkOVOD3_VARKO		SnOVOD2	GCKGHRSCSSLGGRCAKSCKSLGSGYTAYHTCDCARSGLQCCVPHKKG
VkOVOD4_VARKO	LzOVOD14	SnOVOD3	GYRQHCSSYCHGFCSSKCPQHKVAQANCACALYGGICCVS
VkOVOD5_VARKO			GLRDWGSEALLCSGSAPISTADFRRWCWGTLALCHGSYAMEYRRGPFCPPCCL
VkOVOD6_VARKO		SnOVOD5	RHCNTYCYGQCLKSCPYGWVPTSNCACQHTSNICCLPP

Reptile ovodefensin orthologs are also indicated (where they exist) as well as the ovodefensin peptide sequences encoded by each gene

**Table 5:** Physical properties of identified ovodefensin peptides

Defensin domain	Length	Molecular weight	Cys Residue Spacing	Charge	Isoelectric point
VkOVOD1_VARKO	45	4930.77	9–2–4-8-1-4	4	8.0905
VkOVOD2_VARKO	49	5582.3	6–3–13-6	4	8.5149
VkOVOD3_VARKO	48	4950.69	5–6–12–3-1-6	8.5	8.9348
VkOVOD4_VARKO	40	4271.9	3–3–3-9-1-6	4.5	8.0913
VkOVOD5_VARKO	53	5869.76	14–6–13-2	1.5	7.5215
VkOVOD6_VARKO	38	4252.89	3–3–3-9-1-6	3	7.8246

Molecular weights, charges and isoelectric points were calculated based on predicted peptide sequences using the EMBOSS tool pepstats [53]

#### Identification of the Komodo dragon cathelicidin genes

Cathelcidin peptide genes have recently been identified in reptiles through genomics approaches [13]. Several cathelicidin peptide genes have been identified in birds [52, 54–58], snakes [59, 60] and the anole lizard [11, 14, 61]. The release of functional cathelicidin antimicrobial peptides has been observed from chicken heterophils, suggesting that reptilian heterophils may also be a source of these peptides [30, 62]. Alibardi et al. have identified cathelicidin peptides being expressed in anole lizard tissues, including associated with heterophils [11, 14, 61]. Cathelicidin antimicrobial peptides are thought to play key roles in innate immunity in other animals [29] and so likely play this role in the Komodo dragon as well.

In anole lizards, the cathelicidin gene cluster, consisting of 4 genes, is organized as follows: <FASTK> cathelicidin cluster <KLHL18>. We searched for a similar cathelicidin cluster in the Komodo dragon genome. Searching the Komodo dragon genome for cathelicidin-like genes revealed a cluster of three genes that have a “cathelin-like domain”, which is the first requirement of a cathelicidin gene, located at one end of saffold 84. However, this region of scaffold 84 has assembly issues with gaps, isolated exons, and duplications. Identified Komodo dragon cathelicidin genes have been named after their anole orthologs. Two of the Komodo dragon cathelicidins (Cathelicidin2 and Cathelicidin4.1) are in sections with no assembly issues. By contrast, Cathlicidin4.2 was constructed using a diverse set of exons 1–3 and a misplaced exon 4 to create a complete gene, which is paralogous to Cathelicidin4.1. As the cluster is found at one end of the scaffold, there may be additional unidentified cathelicidins that are not captured in this assembly.

A common feature of cathelicidin antimicrobial peptide gene sequences is that the N-terminal cathelin-domain encodes for at least 4 cysteines. In our study of alligator and snake cathelicidins we also noted that typically following the last cysteine, a three-residue pattern consisting of VRR or similar sequence immediately precedes the predicted C-terminal cationic antimicrobial peptide [12, 13, 15, 60, 63]. Additional requirements of a cathelicidin antimicrobial peptide gene sequence are that it encodes for a net-positive charged peptide in the C-terminal region, it is typically encoded by the fourth exon, and it is typically approximately 35 aa in length (range 25–37) [13, 15]. Since the naturally occurring protease responsible for cleavage and release of the functional antimicrobial peptides is not known, prediction of the exact cleavage site is difficult. As can be seen in Table 6**,** the predicted amino acid sequences for each of the identified Komodo dragon cathelicidin gene candidates are listed. Performing our analysis on each sequence, we made predictions and conclusions about whether each potential cathelicidin gene may encode for an antimicrobial peptide.

**Table 6:** Predicted cathelicidin antimicrobial peptide gene sequences

Cathelicidin Gene	Predicted Sequence of Precursor Protein
Cathelicidin2_VARKO	MELFLLGVTLLMLGVPGATSPPLDAPSALLPRDVARMAVEDFNQEAEGQAVFRLLKLKHTHKVKFRWGFHFSLNFTIKETH**C**RKSDNYRIEN**C**RYKTKGPIQD**C**SAQVSVLNFMPDSPLTSVK**C**RRGNGRGSRKAQPSAMLQAEERQPQVHVEVYLPSAYSIAALSVAEEEPSRPAARPH
Cathelicidin4.1_VARKO	MASPWALPLMLLLLGMVGAAPSTAPPSSYDQVIAAAVDIYNQQQKPEYAFRLLEAEPQPDWNSSAQTNQVLRFSIKETV**C**RPSEKADLSQ**C**DYKPDGVDRD**C**SGFYSPQQSPPTIMVQ**C**EDVDQELDR*VTR***FRWRRFFRKAKRFLKRHGVSIAIGTVRLLRRFG**
Cathelicidin4.2_VARKO	MASPRALLLMLLLLRTVGAAPSAAPPSSYEQVIAAAVDTYNQQQKPAYAFRLLEAEPQPDWNPSAGTTQPLRFSIKETV**C**RPSEKADLSQ**C**DYKPNGVDRD**C**SGFYSPQQQSPPTILVQ**C**EDLDR*VTR***RRWRRFFQKAKRFVKRHGVSIAVGAYRIIG**

Full predicted animo acid sequence of Komodo cathelicidin proteins. Note, the Cathelicidin2_VARKO lacks the VTR sequence that is typically present in other reptile cathleicidin precursor proteins, including Cathelicidin4.1_VARKO and Cathelicidin4.2_VARKO

It can be seen that the predicted N-terminal protein sequence of Cathelicidin2_VARKO (VK-CATH2) contains four cysteines (underlined, Table 6). However, there is not an obvious “VRR” or similar sequence in the ~ 10 amino acids following the last cysteine residue as we saw in the alligator and related cathelicidin sequences [12, 13, 15]. In addition, analysis of the 35 C-terminal amino acids reveals a predicted peptide sequence lacking a net positive charge. For these reasons, we predict that the Cathelicidin2_VARKO gene sequence does not encode for an active cathelicidin antimicrobial peptide at its C-terminus **(**Table 7).

**Table 7:** Predicted active cathelicidin peptides and calculated properties (APD3 [64])

Peptide Name	Predicted Functional Cathelicidin Sequence	Length	Net Charge	MW
VK-CATH2	No C-terminal cathelicidin peptide predicted.	NA	NA	NA
VK-CATH4.1	FRWRRFFRKAKRFLKRHGVSIAIGTVRLLRRFG	33 aa	+ 12	4133.02
VK-CATH4.2	RRWRRFFQKAKRFVKRHGVSIAVGAYRIIG	30 aa	+ 10	3660.39

For the identified Cathelicidin4.1_VARKO gene, the predicted cathelin-domain includes the requisite four cysteine residues (Table 6), and the sequence “VTR” is present within 10 amino acids of the last cysteine, similar to the “VRR” sequence in the alligator cathelicidin gene [12, 13, 15]. The 33-aa C-terminal peptide following the “VTR” sequence is predicted to have a net + 12 charge at physiological pH, and a large portion of the sequence is predicted to be helical [65, 66], which is consistent with cathelicidins. The majority of known cathelicidins contain segments with significant helical structure [67]. Finally, analysis of the sequence using the Antimicrobial Peptide Database indicates that the peptide is potentially a cationic antimicrobial peptide [64]. Hence, we predict that this gene likely encodes for an active cathelicidin antimicrobial peptide, called VK-CATH4.1 **(**Table 7**)**.

In addition, this peptide demonstrates some homology to other known antimicrobial peptides in the Antimicrobial Peptide Database [64] (Table 8). It shows a particularly high degree of sequence similarity to cathelicidin peptides identified from squamates, with examples included in Table 8. Thus, the predicted VK-CATH4.1 peptide has many of the hallmark characteristics of a cathelicidin peptide and is a strong candidate for further study. Table 8 shows the alignment of VK_CATH4.1 with known peptides in the Antimicrobial Peptide Database [64].

**Table 8:** Comparison to other cathelicidins

Name	Species	Sequence	Length	VK-CATH4.1/ VK-CATH4.2 Similarity (%)
VK-CATH4.1	*V. komodoensis*	----FRWRRFFRKAKRFLKRHGVSIAIGTVRLLRRFG-	33	100/70.00
VK-CATH4.2	*V. komodoensis*	----RRWRRFFQKAKRFVKRHGVSIAVGAYRIIG----	30	70.00/100.00
Reptiles (Squamates)				
GJ-CATH	*G. japonicus*	-----RWRRFWGKAKRGIKKHGVSIALAALRLRG----	29	62.07/65.52
AC-CATH	*A. carolinensis*	*-----RWGRFWRGAKRFVKKHGVSIALAGLR-------*	26	65.38/65.38
PB-CATH	*P. bivitatis*	-----RWRRFIRGAGRFARRYGWRIALG----------	23	60.87/56.52
NA-CATH	*N. atra*	----KRFKKFFKKLKNSVKKRAKKFFKKPKVIGVTFPF	34	21.21/26.67
Other Vertebrates				
AM-CATH	*A. mississippiensis*	-KIKKGFKKIFKRLPP--IGVGVSIPLAGKR-------	28	24.00/24.00
CAP18	*O. cuniculus*	-GLRKRLRKFRNKIKEKLKKIGQKIQGFVPKLAPRTDY	37	33.33/26.67
SMAP-29	*O. aries*	----RGLRRLGRKIAHGVKKYGPTVL-RIIRIAG----	29	24.14/34.48
cc-CATH	*C. coturnix*	LVQRGRFGRFLKKVRRFIPKVIIAAQIG-----SRFG	32	39.29/28.00
CMAP27	*G. gallus*	-----RFGRFLRKIRRFRPKVTITIQGS-----ARFG-	27	40.74/29.17
Lactoferritin	*B. taurus*	-FKCRRWQWR-------MKKLGAPSITCV---RRAF--	25	27.27/25.00

Homology and alignment of proposed Komodo dragon cathelicidin peptides with known peptides in the Antimicrobial Peptide Database APD3 [64]. These sequence alignments and sequence similarities were generated using Clustal ω [68]

For the identified Cathelicidin4.2_VARKO gene, the predicted cathelin domain includes the requisite four cysteine residues (Table 6). As was noted in the Cathelicidin4.1_VARKO gene, the sequence “VTR” is present within 10 amino acids of the fourth cysteine residue, and immediately precedes the C-terminal segment, which encodes for a 30-aa peptide that is predicted to be antimicrobial [64]. The amino acid sequence of the C-terminal peptide is predicted to have a net + 10 charge at physiological pH, and it demonstrates varied degrees of homology to other known antimicrobial peptides in the Antimicrobial Peptide Database [64]. Thus, like VK-CATH4.1, this candidate peptide also exhibits many of the hallmark characteristics associated with cathelicidin peptides, and is a second strong candidate for further study. Table 8 shows the homology and alignment of VK-CATH4.2 with known peptides from the Antimicrobial Peptide Database. Finally, the gene sequence encoding the functional peptide VK-CATH4.2 is found on exon 4, which is the typical location of the active cathelicidin peptide. This exon encodes the peptide sequence LDRVTRRRWRRFFQKAKRFVKRHGVSIAVGAYRIIG.

The predicted peptide VK-CATH4.2 is highly homologous with peptides from other predicted cathelicidin genes, with similar predicted C-terminal peptides, from *A. carolinensis*, *G. japonicus*, and *P. bivittatus* (Table 8). Residues 2–27 of VK-CATH4.2 are 65% identical and 80% similar to the anole Cathelicidin-2 like predicted C-terminal peptide (XP_008116755.1, aa 130–155). Residues 2–30 of VK-CATH4.2 are 66% identical and 82% similar to the gecko Cathelicidin-related predicted C-terminal peptide (XP_015277841.1, aa 129–151). Finally, aa 2–24 of VK-CATH4.2 are 57% identical and 73% similar to the Cathelicidin-related OH-CATH-Like predicted C-terminal peptide (XP_007445036.1, aa 129–151).

## Conclusions

Reptiles, including Komodo dragons, are evolutionarily ancient, are found in diverse and microbially-challenging environments, and they accordingly appear to have evolved robust innate immune systems. All of these features suggest that reptiles may express interesting antimicrobial peptides. A few reptilian antimicrobial peptides including defensin and cathelicidin peptides have been previously identified and studied that demonstrate broad-spectrum antimicrobial and antifungal activities. While defensins and cathelicidins are known in three of the four orders of reptiles: the testudines, crocodilians, and the squamata, few peptides have been identified to date in lizards and none in varanids (including Komodo dragon).

Genes encoding antimicrobial peptides involved in innate immunity have previously been found in birds and reptiles, some of which are localized within clusters in the genome. Cathelicidin genes have been identified in birds and reptiles, including crocodilians, lizards and snakes. Clusters of β-defensin genes were recently identified in birds by one of our team [52]. While the origins of these gene clusters have not been well established, the phenomenon may have biological significance, potentially helping to coordinate the expression of these genes. Thus, these functionally related loci may have been selectively maintained through reptile and avian innate immunity evolution.

This paper presents a new genome, that of the Komodo dragon, one of the largest extant lizards and the largest vertebrate to exhibit the ability to reproduce through parthenogenesis. Annotated genomes have been published for only a limited number of lizard species, and the present Komodo dragon genome is the first varanid genome assembly to be reported, and therefore will help to expand our understanding of lizard evolution in general. We present an annotated genome that contains as many as 17,213 genes. While there are many aspects of evolution and biology of interest to study in the Komodo dragon, we chose to focus on aspects of innate immunity, specifically antimicrobial peptides, as this was the source of our interest in the Komodo genome [24].

Antimicrobial peptides are present in mammals, birds, amphibians and fish but have not been well-characterized in reptiles despite the central position of this class in vertebrate evolution. We have sought to contribute to this understanding through our prior studies of antimicrobial peptides from birds [52], alligators [12, 21–23], snakes [12, 60, 63, 69–72], and now Komodo dragon [24, 25].

In the present study, we report the identification of genes encoding Komodo dragon defensin and cathelicidin peptides. We have elucidated 66 potential β–defensin genes, including 18 that appear to be unique to Komodo dragons. The remaining 48 peptides appear to have homologs in anole lizards and/or snakes. Similar to avian genomes, the Komodo dragon genome does not contain α-defensin genes; this class of antimicrobial peptides appears to be restricted to mammals [13]. Additionally, six potential β-ovodefensins were identified in the genome. These β–defensin and β-ovodefensin genes are localized in defensin-gene clusters within the genome.

In addition to defensins, we identified three potential cathelicidin genes in the genome; however, upon further analysis it was determined that one of these apparent cathelicidin genes did not actually encode a cathelicidin peptide. The remaining two genes, Cathelicidin4.1_VARKO and Cathelicidin4.2_VARKO, are predicted to encode functional cathelicidin peptides at the C-terminal end of the precursor peptide. These peptides show significant degrees of similarity to other reptile cathelicidins. These findings are significant; however, the identified defensin and cathelicidin gene clusters appear to reside near scaffold edges, and therefore may not represent the full complement of defensin and cathelicidin genes that may be present in the Komodo dragon genome.

The defensin and cathelicidin genes and gene clusters that we have identified here exhibit similarities to those that have been reported for the anole lizard and snakes, but they also show characteristics that are unique to the Komodo dragon. We anticipated that the findings presented here should contribute to a deeper understanding of innate immunity and antimicrobial peptides in reptiles and vertebrates in general.

## Methods & experimental procedures

### Komodo dragon blood samples

Komodo dragon (*Varanus komodoensis*) blood was collected by staff at the St. Augustine’s Alligator Farm Zoological Park (St. Augustine, FL) in compliance with relevant guidelines, using protocols approved by the GMU IACUC (GMU IACUC# 0266). Blood was collected in plastic blood collecting tubes treated with K_2_EDTA as the anticoagulant. Samples were immediately placed on ice, and then shipped on ice overnight to GMU.

### Library preparation and multiplexing

Genomic DNA was prepared from a sample that had been enriched for leukocytes by a settling protocol (24 h, 37 °C, 5% CO_2_) from fresh Komodo dragon blood. DNA-seq libraries were constructed using PrepX ILM DNA Library Reagent Kit (Catalog No. 400044, Lot No. F0199) on the Apollo 324 robot (WaferGen, CA). Briefly, 150 ng of genomic DNA was resuspended in 50 μl of nuclease-free water and fragmented to 200–250 bp, using Covaris M220 to 300 bp at Peak Incident Power of (W) 50, Duty Factor of 20%, Cycles per Burst of 200, and Treatment Time of 75 s. Briefly, the ends were repaired and an ‘A’ base added to the 3′ end, preparing the DNA fragments for ligation to the adapters, which have a single ‘T’ base overhang at their 3′ end. The adapters enabled PCR amplification and hybridization to the flow cell. Following ligation, the excess adapters were removed and 300 ± 50 bp fragments (225 bp insert) were enriched for library amplification by PCR. The library that was generated was then validated using an Agilent 2100 Bioanalyzer and quantitated using a Quant-iT dsDNA HS Kit (Invitrogen) and qPCR. The samples were multiplexed based on qPCR quantitation to obtain similar distribution of reads of multiplexed samples.

### Chicago library preparation

High molecular weight genomic DNA was extracted from blood cells collected from fresh Komodo dragon whole blood. A Chicago library was prepared as described previously [73]. Briefly, ≥ 0.5 μg of high molecular weight genomic DNA (50 kbp mean fragment size) was extracted from whole Komodo dragon blood using a Qiagen blood and cell midi kit, reconstituted into chromatin in vitro, and fixed with formaldehyde. Fixed chromatin was then digested with MboI, the 5′ overhangs were filled in with biotinylated nucleotides, and then free blunt ends were ligated. After ligation, crosslinks were reversed and the DNA purified from protein. Purified DNA was treated to remove biotin that was not internal to ligated fragments. The DNA was sheared to ~ 350 bp mean fragment size, and sequencing libraries were generated using NEBNext Ultra enzymes and Illumina-compatible adapters. Biotin-containing fragments were then isolated using streptavidin beads before PCR enrichment of the library.

### Cluster generation and HiSeq paired-end sequencing

Libraries were clustered onto a flow cell using Illumina’s TruSeq PE Cluster Kit v3-cBOT-HS (PE-401-3001) and sequenced on an Illumina HiSeq 2500. The Chicago library was sequenced using 2 × 101 PE Rapid-Run (153 M read pairs) and the TruSeq SBS Kit v3-HS (200-cycles) (FC-401-3001), while the Virginia Bioinformatics Institute Genomics Core provided a 2 × 151 PE Rapid-Run (149 M read pairs) using TruSeq Rapid SBS Kit-200 cycle (2500) (FC-402–4001) and two TruSeq Rapid SBS Kit-50 cycles (FC-402–4002).

### Scaffolding the draft genome with HiRise

N50 is defined as the scaffold length such that the sum of the lengths of all scaffolds of this size or less is equal to 50% of the total assembly length. The initial Komodo dragon draft genome assembly in FASTA format generated at Virginia Tech with Illumina 150 PE (Celera Assembler 8.2, default parameters, [74]) resulted in 1599 Mbp with a scaffold N50 of 35.8 kbp. This assembly, additional Illumina shotgun sequences (100 PE) and Chicago library sequence in FASTQ format were used as input data for HiRise, a software pipeline designed specifically for using Chicago library sequence data to assemble genomes [73]. Shotgun and Chicago library sequences were aligned to the draft input assembly using a modified SNAP read mapper (http://snap.cs.berkeley.edu). The separations of Chicago read pairs mapped within draft scaffolds were analyzed by HiRise to produce a likelihood model, and the resulting likelihood model was used to identify putative misjoins and score prospective joins. After scaffolding, shotgun sequences were used to close gaps between contigs.

### Genome annotation and completeness

Assembly sequences were first masked using RepeatMasker (v4.0.3, http://www.repeatmasker.org/) with parameters set to “-s -a -nolow” and using a customized repeat library. Protein-coding genes were predicted using MAKER2 [75], which used anole lizard (*A. carolinensis*, version AnoCar2.0) and python (*P. bivittatus*, version bivittatus-5.0.2) protein sequences that were downloaded from Ensembl (www.ensembl.org) and RefSeq (www.ncbi.nlm.nih.gov/refseq) as protein homology evidence, along with the previously assembled RNA-seq data [24] as the expression evidence, and integrated with prediction methods including Blastx, SNAP [76] and Augustus [77]. The SNAP HMM file was generated by training the anole lizard gene sequences. An Augustus model file was generated by training 3026 core genes of vertebrates from a genome completeness assessment tool BUSCO [78]. Predicted genes were subsequently used as query sequences in a Blastx database search of NR database (the non-redundant database, http://www.ncbi.nlm.nih.gov/). Blastx alignments with e-value greater than 1e− 10 were discarded, and the top hit was used to annotate the query genes. Repeat families were identified by using the de novo modeling package RepeatModeler (http://www.repeatmasker.org/RepeatModeler/). Then, the de novo identified repeat sequences were combined with manually selected vertebrate repeats from RepBase (https://www.girinst.org/repbase/) to form a customized repeat library. The completeness of assembly was estimated using CEGMA by examining 248 core eukaryotic genes [35].

### Transcriptome

A transcriptome generated from RNA isolated from Komodo blood cells has been previously described [24] and was used here to aid in the assembly annotation. Briefly, 280–300 bp libraries (160–180 bp insert) were generated, clustered onto a flow cell using Illumina’s TruSeq PE Cluster Kit v3-cBOT-HS and sequenced using TruSeq SBS Kit v3-HS (300 cycles, 2 × 150 cycle paired-end) on an Illumina HiSeq 2500.

### Identification of defensin and cathelicidin genes within the genome

Lizard and snake defensin and cathelicidin genes had been previously identified in prior analyses of published genomes for *Anolis carolinensis* [34] *Ophiophagus hannah* (king cobra) [79] *Python bivittatus* (Burmese python) [80] as well as the pit vipers *Protobothrops mucrosquamatus* (https://www.ncbi.nlm.nih.gov/genome/annotation_euk/Protobothrops_mucrosquamatus/100/) and *Vipera berus berus* (https://www.ncbi.nlm.nih.gov/bioproject/170536) (https://www.hgsc.bcm.edu/reptiles/european-adder-genome-project) (Additional file 3: Table S2). This data was used in our analyses of the Komodo dragon genome. Genes from *A. carolinensis* (β-defensins, ovodefensins, cathelicidins, and genes flanking the defensin and cathelicidin clusters) were used as queries in a TBLASTN against the Komodo genome. Due to the diversity of β-defensins, homology searches are not sufficient to identify the entire β-defensin repertoire, so a combination of strategies was used. Genomic scaffolds containing hits were extracted and genes identified by BLAST were manually curated using Artemis [19]. Scaffolds with hits to β-defensins were then further examined manually for the characteristic β-defensin motif and signal peptides not previously identified by the initial BLAST search. Gene structures were determined based on previously annotated *A. carolinensis* orthologs when possible.

Annotated β-defensin genes were named by using the initials for the species and genus (Vk) as a prefix and a five-letter abbreviation as a suffix (VkBDx_VARKO) and numbered in order following CTSB on scaffold 210. Β-ovodefensins were similarly named in order following MTMR9 (VkOVODx_VARKO). Β-defensins on scaffold 826 were numbered using anole orthologs as a reference for gene order. Β-defensins on other scaffolds were named based on their anole orthologs. Cathelicidins were named based on their anole orthologs.

### Peptide prediction

Predicted amino acid sequences were compared to other known protein sequences using blast-p at NCBI (https://www.ncbi.nlm.nih.gov) tool [81, 82]. Prediction of size, charge, helicity and other properties of proposed antimicrobial peptides was performed using Antimicrobial Peptide Database APD3 Calculation and Prediction tool http://aps.unmc.edu/AP/prediction/prediction_main.php [64]. Homology searching against other peptides in the APD3 database was done using the proffered option after the calculation and prediction tool was applied.

## Additional files

Additional file 1: **Figure S1.** Repeat families. The Komodo dragon genomic profile of repeat element families. (JPG 662 kb)

Additional file 2: **Table S1.** Repeat element families. (DOCX 61 kb)

Additional file 3: **Table S2.** Reptile Defensin Orthologs. (DOCX 158 kb)

## Data Availability

Genomic data are available at NCBI with raw sequencing reads deposited in the Sequence Read Archive (accession #SRP161190), while the genome assembly has been deposited at DDBJ/ENA/GenBank under the accession VEXN00000000. The assembly version described in this paper is VEXN01000000.

## Abbreviations

aa: Amino acid
BLK: Proto-oncogene, Src family tyrosine kinase
bp: Base pair
CTSB: Cathepsin B gene
DNA: Deoxyribonucleic acids
DNA-seq: DNA sequencing
FAM167A: Family with sequence similarity 167, member A
FASTK: Fas Activated Serine/ Threonine Kinase
FDFT1: Farnesyl-diphosphate farnesyl transferase 1
Gb: Gigabase
GMU: George Mason University
Hrs: Hours
IACUC: Institutional Animal Care and Use Committee
kbp: Kilo base pair
KLHL18: Kelch Like Family Member 18
Mbp: Mega base pairs
MTMR9: Myotubularin related protein 9
PCR: Polymerase chain reaction
qPCR: Quantitative polymerase chain reaction
RNA: Ribonucleic acid
RNA-seq: RNA sequencing
SNAP: Scalable Nucleotide Alignment Program
TRAM2: Translocation associated membrane protein 2
XKR6: XK, Kell blood group complex subunit-related family, member 6
XPO1: Exportin 1
μg: Microgram
μl: Microliter

## References

[1] Hocknull SA, Piper PJ, van den Bergh GD, Due RA, Morwood MJ, Kurniawan I (2009). Dragon's paradise lost: palaeobiogeography, evolution and extinction of the largest-ever terrestrial lizards (Varanidae). PLoS One.

[2] Centre WCM (1996). IUCN Red List of Threatened Species 2015.

[3] Bull J. J., Jessop Tim S., Whiteley Marvin (2010). Deathly Drool: Evolutionary and Ecological Basis of Septic Bacteria in Komodo Dragon Mouths. PLoS ONE.

[4] Montgomery Joel M., Gillespie Don, Sastrawan Putra, Fredeking Terry M., Stewart George L. (2002). AEROBIC SALIVARY BACTERIA IN WILD AND CAPTIVE KOMODO DRAGONS. Journal of Wildlife Diseases.

[5] Goldstein EJ, Tyrrell KL, Citron DM, Cox CR, Recchio IM, Okimoto B, Bryja J, Fry BG (2013). Anaerobic and aerobic bacteriology of the saliva and gingiva from 16 captive Komodo dragons (*Varanus komodoensis*): new implications for the "bacteria as venom" model. J Zoo Wildl Med.

[6] Merchant Mark, Henry Danyell, Falconi Rodolfo, Muscher Bekky, Bryja Judith (2013). Antibacterial activities of serum from the Komodo Dragon (Varanus komodoensis). Microbiology Research.

[7] Zimmerman LM, Vogel LA, Bowden RM (2010). Understanding the vertebrate immune system: insights from the reptilian perspective. J Exp Biol.

[8] Findlay F, Proudfoot L, Stevens C, Barlow PG (2016). Cationic host defense peptides; novel antimicrobial therapeutics against category a pathogens and emerging infections. Pathog Glob Health.

[9] Haney EF, Straus SK, Hancock REW (2019). Reassessing the host defense peptide landscape. Front Chem.

[10] Alibardi L, Celeghin A, Dalla Valle L (2012). Wounding in lizards results in the release of beta-defensins at the wound site and formation of an antimicrobial barrier. Dev Comp Immunol.

[11] Alibardi L (2015). Immunocytochemical detection of beta-defensins and cathelicidins in the secretory granules of the tongue in the lizard Anolis carolinensis. Acta Histochem.

[12] Barksdale SM, Hrifko EJ, van Hoek ML (2017). Cathelicidin antimicrobial peptide from Alligator mississippiensis has antibacterial activity against multi-drug resistant Acinetobacter baumanii and Klebsiella pneumoniae. Dev Comp Immunol.

[13] van Hoek ML (2014). Antimicrobial peptides in reptiles. Pharmaceuticals (Basel).

[14] Alibardi L (2014). Ultrastructural immunolocalization of chatelicidin-like peptides in granulocytes of normal and regenerating lizard tissues. Acta Histochem.

[15] van Hoek ML, Epand RM (2016). Diversity in Host Defense Antimicrobial Peptides. Host Defense Peptides and Their Potential as Therapeutic Agents.

[16] Merchant ME, Leger N, Jerkins E, Mills K, Pallansch MB, Paulman RL, Ptak RG (2006). Broad spectrum antimicrobial activity of leukocyte extracts from the American alligator (Alligator mississippiensis). Vet Immunol Immunopathol.

[17] Merchant ME, Mills K, Leger N, Jerkins E, Vliet KA, McDaniel N (2006). Comparisons of innate immune activity of all known living crocodylian species. Comp Biochem Physiol B Biochem Mol Biol.

[18] Merchant ME, Pallansch M, Paulman RL, Wells JB, Nalca A, Ptak R (2005). Antiviral activity of serum from the American alligator (Alligator mississippiensis). Antivir Res.

[19] Merchant ME, Roche C, Elsey RM, Prudhomme J (2003). Antibacterial properties of serum from the American alligator (Alligator mississippiensis). Comp Biochem Physiol B Biochem Mol Biol.

[20] Kommanee J, Preecharram S, Daduang S, Temsiripong Y, Dhiravisit A, Yamada Y, Thammasirirak S (2012). Antibacterial activity of plasma from crocodile (Crocodylus siamensis) against pathogenic bacteria. Ann Clin Microbiol Antimicrob.

[21] Barksdale SM, Hrifko EJ, Chung EM, van Hoek ML (2016). Peptides from American alligator plasma are antimicrobial against multi-drug resistant bacterial pathogens including Acinetobacter baumannii. BMC Microbiol.

[22] Bishop BM, Juba ML, Devine MC, Barksdale SM, Rodriguez CA, Chung MC, Russo PS, Vliet KA, Schnur JM, van Hoek ML (2015). Bioprospecting the American Alligator (Alligator mississippiensis) host defense Peptidome. PLoS One.

[23] Juba ML, Russo PS, Devine M, Barksdale S, Rodriguez C, Vliet KA, Schnur JM, van Hoek ML, Bishop BM (2015). Large scale discovery and De novo-assisted sequencing of cationic antimicrobial peptides (CAMPs) by microparticle capture and electron-transfer dissociation (ETD) mass spectrometry. J Proteome Res.

[24] Bishop BM, Juba ML, Russo PS, Devine M, Barksdale SM, Scott S, Settlage R, Michalak P, Gupta K, Vliet K (2017). Discovery of novel antimicrobial peptides from *Varanus komodoensis* (Komodo dragon) by large-scale analyses and De-novo-assisted sequencing using electron-transfer dissociation mass spectrometry. J Proteome Res.

[25] Chung EMC, Dean SN, Propst CN, Bishop BM, van Hoek ML (2017). Komodo dragon-inspired synthetic peptide DRGN-1 promotes wound-healing of a mixed-biofilm infected wound. NPJ Biofilms Microbiomes.

[26] Whenham N, Lu TC, Maidin MB, Wilson PW, Bain MM, Stevenson ML, Stevens MP, Bedford MR, Dunn IC (2015). Ovodefensins, an oviduct-specific antimicrobial gene family, Have Evolved in Birds and Reptiles to Protect the Egg by Both Sequence and Intra-Six-Cysteine Sequence Motif Spacing. Biol Reprod.

[27] Ganz T (2004). Defensins: antimicrobial peptides of vertebrates. C R Biol.

[28] Bals R, Wilson JM (2003). Cathelicidins--a family of multifunctional antimicrobial peptides. Cell Mol Life Sci.

[29] van Harten Roel, van Woudenbergh Esther, van Dijk Albert, Haagsman Henk (2018). Cathelicidins: Immunomodulatory Antimicrobials. Vaccines.

[30] van Dijk A, Tersteeg-Zijderveld MH, Tjeerdsma-van Bokhoven JL, Jansman AJ, Veldhuizen EJ, Haagsman HP (2009). Chicken heterophils are recruited to the site of Salmonella infection and release antibacterial mature Cathelicidin-2 upon stimulation with LPS. Mol Immunol.

[31] Molhoek EM, van Dijk A, Veldhuizen EJ, Dijk-Knijnenburg H, Mars-Groenendijk RH, Boele LC, Kaman-van Zanten WE, Haagsman HP, Bikker FJ (2010). Chicken cathelicidin-2-derived peptides with enhanced immunomodulatory and antibacterial activities against biological warfare agents. Int J Antimicrob Agents.

[32] Schneider VAF, Coorens M, Tjeerdsma-van Bokhoven JLM, Posthuma G, van Dijk A, Veldhuizen EJA, Haagsman HP. Imaging the Antistaphylococcal Activity of CATH-2: Mechanism of Attack and Regulation of Inflammatory Response. mSphere. 2017;2:e00370–17. 10.1128/mSphere.00370-17 PMC5663982 29104934

[33] Gregory TR. Animal genome size database. 2019. [http://www.genomesize.com].

[34] Alfoldi J, Di Palma F, Grabherr M, Williams C, Kong L, Mauceli E, Russell P, Lowe CB, Glor RE, Jaffe JD (2011). The genome of the green anole lizard and a comparative analysis with birds and mammals. Nature.

[35] Parra G, Bradnam K, Korf I (2007). CEGMA: a pipeline to accurately annotate core genes in eukaryotic genomes. Bioinformatics.

[36] Scheetz T, Bartlett JA, Walters JD, Schutte BC, Casavant TL, McCray PB (2002). Genomics-based approaches to gene discovery in innate immunity. Immunol Rev.

[37] Hollox Edward J., Abujaber Razan (2017). Evolution and Diversity of Defensins in Vertebrates. Evolutionary Biology: Self/Nonself Evolution, Species and Complex Traits Evolution, Methods and Concepts.

[38] Ganz T (2003). Defensins: antimicrobial peptides of innate immunity. Nat Rev Immunol.

[39] Semple F, Dorin JR (2012). beta-Defensins: multifunctional modulators of infection, inflammation and more?. J Innate Immun.

[40] Schutte BC, McCray PB (2002). [beta]-defensins in lung host defense. Annu Rev Physiol.

[41] Alibardi L (2013). Ultrastructural immunolocalization of beta-defensin-27 in granulocytes of the dermis and wound epidermis of lizard suggests they contribute to the anti-microbial skin barrier. Anat Cell Biol.

[42] Alibardi L (2013). Granulocytes of reptilian sauropsids contain beta-defensin-like peptides: a comparative ultrastructural survey. J Morphol.

[43] Alibardi L (2014). Histochemical, biochemical and cell biological aspects of tail regeneration in lizard, an amniote model for studies on tissue regeneration. Prog Histochem Cytochem.

[44] Dalla Valle L, Benato F, Maistro S, Quinzani S, Alibardi L (2012). Bioinformatic and molecular characterization of beta-defensins-like peptides isolated from the green lizard Anolis carolinensis. Dev Comp Immunol.

[45] Zhu S, Gao B (2013). Evolutionary origin of beta-defensins. Dev Comp Immunol.

[46] Zhang G, Sunkara LT (2014). Avian antimicrobial host defense peptides: from biology to therapeutic applications. Pharmaceuticals (Basel).

[47] Schutte BC, Mitros JP, Bartlett JA, Walters JD, Jia HP, Welsh MJ, Casavant TL, McCray PB (2002). Discovery of five conserved beta -defensin gene clusters using a computational search strategy. Proc Natl Acad Sci U S A.

[48] Jia HP, Schutte BC, Schudy A, Linzmeier R, Guthmiller JM, Johnson GK, Tack BF, Mitros JP, Rosenthal A, Ganz T, McCray PB (2001). Discovery of new human beta-defensins using a genomics-based approach. Gene.

[49] Huang Y, Li Y, Burt DW, Chen H, Zhang Y, Qian W, Kim H, Gan S, Zhao Y, Li J (2013). The duck genome and transcriptome provide insight into an avian influenza virus reservoir species. Nat Genet.

[50] Hellgren O, Ekblom R (2010). Evolution of a cluster of innate immune genes (beta-defensins) along the ancestral lines of chicken and zebra finch. Immunome Res.

[51] Xiao Y, Hughes AL, Ando J, Matsuda Y, Cheng JF, Skinner-Noble D, Zhang G (2004). A genome-wide screen identifies a single beta-defensin gene cluster in the chicken: implications for the origin and evolution of mammalian defensins. BMC Genomics.

[52] Cheng Y, Prickett MD, Gutowska W, Kuo R, Belov K, Burt DW (2015). Evolution of the avian beta-defensin and cathelicidin genes. BMC Evol Biol.

[53] Rice P, Longden I, Bleasby A (2000). EMBOSS: the European molecular biology open software suite. Trends Genet.

[54] van Dijk A, Molhoek EM, Veldhuizen EJ, Bokhoven JL, Wagendorp E, Bikker F, Haagsman HP (2009). Identification of chicken cathelicidin-2 core elements involved in antibacterial and immunomodulatory activities. Mol Immunol.

[55] Xiao Y, Cai Y, Bommineni YR, Fernando SC, Prakash O, Gilliland SE, Zhang G (2006). Identification and functional characterization of three chicken cathelicidins with potent antimicrobial activity. J Biol Chem.

[56] van Dijk A, Veldhuizen EJ, van Asten AJ, Haagsman HP (2005). CMAP27, a novel chicken cathelicidin-like antimicrobial protein. Vet Immunol Immunopathol.

[57] Cuperus T, van Dijk A, Dwars RM, Haagsman HP (2016). Localization and developmental expression of two chicken host defense peptides: cathelicidin-2 and avian beta-defensin 9. Dev Comp Immunol.

[58] Feng F, Chen C, Zhu W, He W, Guang H, Li Z, Wang D, Liu J, Chen M, Wang Y, Yu H (2011). Gene cloning, expression and characterization of avian cathelicidin orthologs, cc-CATHs, from Coturnix coturnix. FEBS J.

[59] Wang Y, Hong J, Liu X, Yang H, Liu R, Wu J, Wang A, Lin D, Lai R (2008). Snake cathelicidin from Bungarus fasciatus is a potent peptide antibiotics. PLoS One.

[60] Blower RJ, Barksdale SM, van Hoek ML (2015). Snake cathelicidin NA-CATH and Smaller helical antimicrobial peptides are effective against Burkholderia thailandensis. PLoS Negl Trop Dis.

[61] Dalla Valle L, Benato F, Paccanaro MC, Alibardi L (2013). Bioinformatic and molecular characterization of cathelicidin-like peptides isolated from the green lizard *Anolis carolinensis* (Reptilia: Lepidosauria: Iguanidae). Eur Zool J (Ital J Zool).

[62] Genovese KJ, He H, Swaggerty CL, Kogut MH (2013). The avian heterophil. Dev Comp Immunol.

[63] Blower RJ, Popov SG, van Hoek ML (2017). Cathelicidin peptide rescues G. mellonella infected with B. anthracis. Virulence.

[64] Wang G, Li X, Wang Z (2016). APD3: the antimicrobial peptide database as a tool for research and education. Nucleic Acids Res.

[65] Yang J, Yan R, Roy A, Xu D, Poisson J, Zhang Y (2015). The I-TASSER suite: protein structure and function prediction. Nat Methods.

[66] Roy A, Kucukural A, Zhang Y (2010). I-TASSER: a unified platform for automated protein structure and function prediction. Nat Protoc.

[67] Sorensen OE, Borregaard N (2005). Cathelicidins--nature's attempt at combinatorial chemistry. Comb Chem High Throughput Screen.

[68] Sievers F, Wilm A, Dineen D, Gibson TJ, Karplus K, Li W, Lopez R, McWilliam H, Remmert M, Soding J (2011). Fast, scalable generation of high-quality protein multiple sequence alignments using Clustal omega. Mol Syst Biol.

[69] de Latour Frank A., Amer Lilian S., Papanstasiou Emilios A., Bishop Barney M., Hoek Monique L. van (2010). Antimicrobial activity of the Naja atra cathelicidin and related small peptides. Biochemical and Biophysical Research Communications.

[70] Dean SN, Bishop BM, van Hoek ML (2011). Susceptibility of Pseudomonas aeruginosa biofilm to alpha-helical peptides: D-enantiomer of LL-37. Front Microbiol.

[71] Dean SN, Bishop BM, van Hoek ML (2011). Natural and synthetic cathelicidin peptides with anti-microbial and anti-biofilm activity against Staphylococcus aureus. BMC Microbiol.

[72] Gupta K, Singh S, van Hoek ML (2015). Short, synthetic cationic peptides have antibacterial activity against Mycobacterium smegmatis by forming pores in membrane and synergizing with antibiotics. Antibiotics (Basel).

[73] Putnam NH, O'Connell BL, Stites JC, Rice BJ, Blanchette M, Calef R, Troll CJ, Fields A, Hartley PD, Sugnet CW (2016). Chromosome-scale shotgun assembly using an in vitro method for long-range linkage. Genome Res.

[74] Myers EW, Sutton GG, Delcher AL, Dew IM, Fasulo DP, Flanigan MJ, Kravitz SA, Mobarry CM, Reinert KH, Remington KA (2000). A whole-genome assembly of drosophila. Science.

[75] Holt C, Yandell M (2011). MAKER2: an annotation pipeline and genome-database management tool for second-generation genome projects. BMC Bioinformatics.

[76] Korf I (2004). Gene finding in novel genomes. BMC Bioinformatics.

[77] Stanke M, Waack S (2003). Gene prediction with a hidden Markov model and a new intron submodel. Bioinformatics.

[78] Simao FA, Waterhouse RM, Ioannidis P, Kriventseva EV, Zdobnov EM (2015). BUSCO: assessing genome assembly and annotation completeness with single-copy orthologs. Bioinformatics.

[79] Vonk FJ, Casewell NR, Henkel CV, Heimberg AM, Jansen HJ, McCleary RJ, Kerkkamp HM, Vos RA, Guerreiro I, Calvete JJ (2013). The king cobra genome reveals dynamic gene evolution and adaptation in the snake venom system. Proc Natl Acad Sci U S A.

[80] Castoe TA, de Koning AP, Hall KT, Card DC, Schield DR, Fujita MK, Ruggiero RP, Degner JF, Daza JM, Gu W (2013). The Burmese python genome reveals the molecular basis for extreme adaptation in snakes. Proc Natl Acad Sci U S A.

[81] Altschul SF, Madden TL, Schaffer AA, Zhang J, Zhang Z, Miller W, Lipman DJ (1997). Gapped BLAST and PSI-BLAST: a new generation of protein database search programs. Nucleic Acids Res.

[82] Altschul SF, Wootton JC, Gertz EM, Agarwala R, Morgulis A, Schaffer AA, Yu YK (2005). Protein database searches using compositionally adjusted substitution matrices. FEBS J.

