# Supplementary figures and images for: The Komodo dragon (Varanus komodoensis) genome and identification of innate immunity genes and clusters
Source: BMC Genomics. 2019 Aug 30;20:684. doi: 10.1186/s12864-019-6029-y (PMC6716921; doi:10.1186/s12864-019-6029-y)

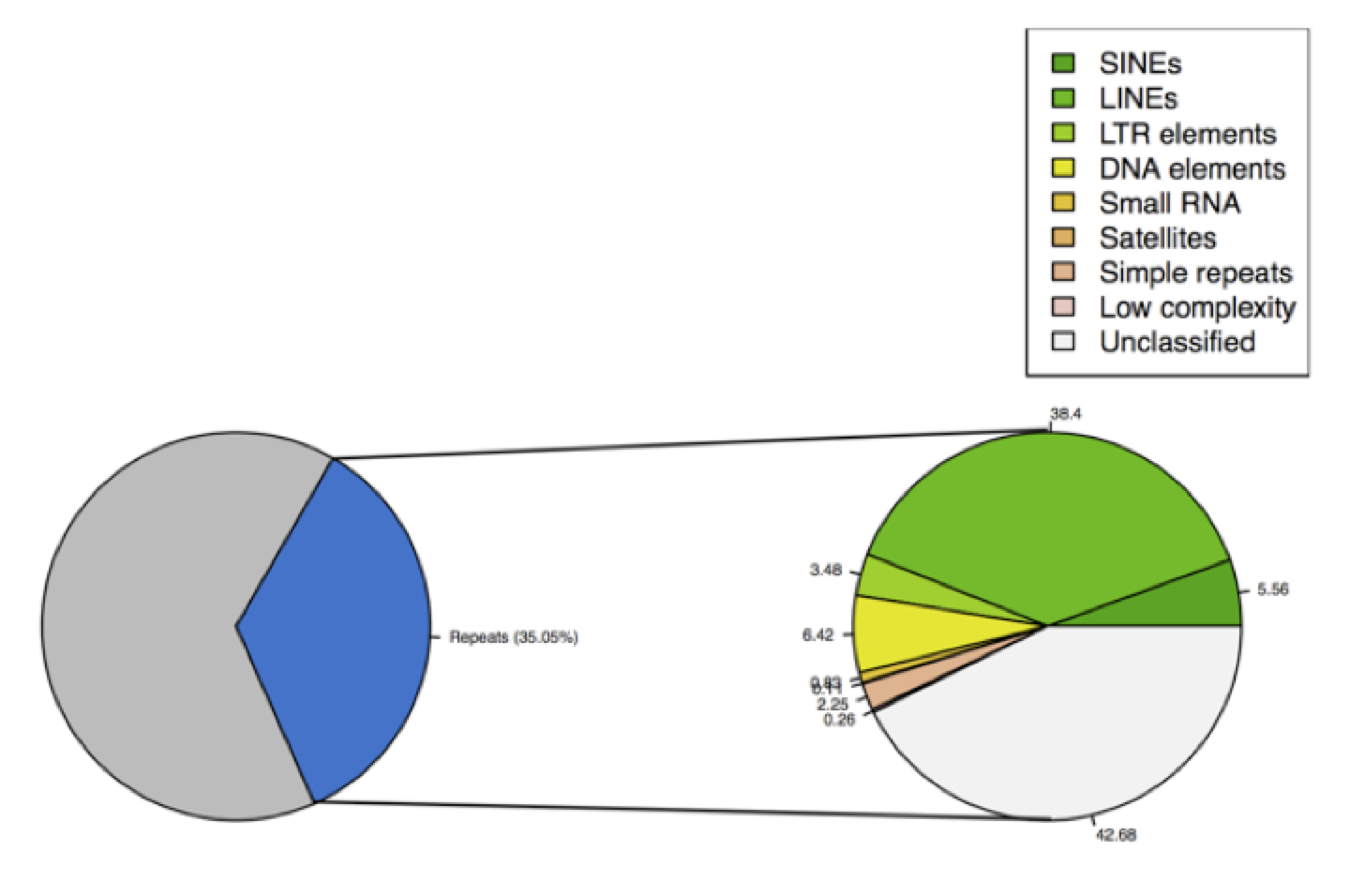

Supplement: Supplementary file 1 — Figure S1. Repeat families. The Komodo dragon genomic profile of repeat element families. (JPG 662 kb) [file 12864_2019_6029_MOESM1_ESM.jpg]
