# Supplementary material for: The Komodo dragon (Varanus komodoensis) genome and identification of innate immunity genes and clusters
Source: BMC Genomics. 2019 Aug 30;20:684. doi: 10.1186/s12864-019-6029-y (PMC6716921; doi:10.1186/s12864-019-6029-y)
Supplement: Supplementary file 2 — Table S1. Repeat element families. (DOCX 61 kb) [file 12864_2019_6029_MOESM2_ESM.docx]

**Supplementary Table S1.**

**Repeat element families.**

| **Family** | **No. of elements** | **Length (bp)** | **Percentage of assembly** |
| --- | --- | --- | --- |
| **SINEs** | 202688 | 31281631 | 1.95 |
| **LINEs** | 618407 | 216156399 | 13.46 |
| **LTR elements** | 32120 | 19631234 | 1.22 |
| **DNA elements** | 221140 | 36128298 | 2.25 |
| **Small RNA** | 44707 | 4616928 | 0.29 |
| **Satellites** | 1285 | 713779 | 0.04 |
| **Simple repeats** | 347432 | 12637550 | 0.79 |
| **Low complexity** | 31736 | 1434326 | 0.09 |
| **Unclassified** | 1068553 | 235967796 | 14.69 |
