# Supplementary material for: The Komodo dragon (Varanus komodoensis) genome and identification of innate immunity genes and clusters
Source: BMC Genomics. 2019 Aug 30;20:684. doi: 10.1186/s12864-019-6029-y (PMC6716921; doi:10.1186/s12864-019-6029-y)
Supplement: Supplementary file 3 — Table S2. Reptile Defensin Orthologs. (DOCX 158 kb) [file 12864_2019_6029_MOESM3_ESM.docx]

**Supplementary Table S2**

**Reptile Defensin Orthologs**

| **Species: *Anolis carolinensis*** | | **Abbreviation: ANOCA** | | | | | |
| --- | --- | --- | --- | --- | --- | --- | --- |
| **Gene** | **Scaffold** | **Coordinates** | | | | **Strand** | |
| LzBD80 | Chromosome 1 | 139941994 | | 139943722 | | forward | |
| LzBD82 | Chromosome 1 | 259462644 | | 259468791 | | forward | |
| LzBD1 | Scaffold 343309 | 253147 | | 255816 | | reverse | |
| LzBD2 | Scaffold 343309 | 267960 | | 270234 | | reverse | |
| LzBD3 | Scaffold 343309 | 274511 | | 277652 | | reverse | |
| LzBD4 | Scaffold 343309 | 280019 | | 284959 | | reverse | |
| LzBD5 | Scaffold 343309 | 304587 | | 305780 | | reverse | |
| LzBD6 | Scaffold 343309 | 326250 | | 328443 | | forward | |
| LzBD11 | Scaffold 343309 | 453777 | | 456975 | | forward | |
| LzBD12 | Scaffold 343309 | 474902 | | 478230 | | forward | |
| LzBD13 | Scaffold 343309 | 484393 | | 487366 | | forward | |
| LzBD14 | Scaffold 343309 | 491657 | | 493344 | | reverse | |
| LzBD15 | Scaffold 343309 | 515527 | | 516551 | | reverse | |
| LzBD16 | Scaffold 343309 | 560065 | | 561167 | | forward | |
| LzBD17 | Scaffold 343309 | 597499 | | 599023 | | reverse | |
| LzBD26 | Scaffold 343309 | 713535 | | 715336 | | reverse | |
| LzBD27 | Scaffold 343309 | 730122 | | 734051 | | forward | |
| LzBD31 | Scaffold 343309 | 821588 | | 821718 | | reverse | |
| LzBD32 | Scaffold 343309 | 838829 | | 844257 | | forward | |
| LzBD33 | Scaffold 343309 | 854065 | | 857051 | | forward | |
| LzBD34 | Scaffold 343309 | 864678 | | 866740 | | forward | |
| LzBD35 | Scaffold 343309 | 874550 | | 879602 | | reverse | |
| LzBD37 | Scaffold 343309 | 896777 | | 900492 | | reverse | |
| LzBD39 | Scaffold 343309 | 934953 | | 939357 | | forward | |
| LzBD41 | Scaffold 343309 | 966042 | | 967538 | | reverse | |
| LzBD43 | Scaffold 343309 | 978703 | | 980206 | | forward | |
| LzBD50 | Scaffold 343309 | 1086778 | | 1088325 | | forward | |
| LzBD52 | Scaffold 343309 | 1112320 | | 1119102 | | reverse | |
| LzBD53 | Scaffold 343309 | 1140842 | | 1145769 | | reverse | |
| LzBD54 | Scaffold 343309 | 1157327 | | 1159293 | | reverse | |
| LzBD55 | Scaffold 343309 | 1166685 | | 1172305 | | reverse | |
| LzBD57 | Scaffold 343309 | 1205853 | | 1205974 | | reverse | |
| LzBD58 | Scaffold 343309 | 1237094 | | 1240294 | | reverse | |
| LzBD63 | Scaffold 343309 | 1299517 | | 1304767 | | reverse | |
| LzBD77 | Scaffold 343523 | 32168 | | 35434 | | forward | |
| LzBD78 | Scaffold 343523 | 40383 | | 48103 | | forward | |
| LzBD81 | Scaffold 343551 | 279957 | | 282163 | | forward | |
| LzOVOD14 | Scaffold 343551 | 17205 | | 17324 | | reverse | |
| LzOVOD1 | Scaffold 343551 | 200316 | | 200458 | | reverse | |
|  |  |  | | |  | | |
| **Species: *Ophiophagus hanna*** | | **Abbreviation: OPHHA** | | | | | |
| **Gene** | **Scaffold** | **Coordinates** | | | | **Strand** | |
| SnBD40_OPHHA | Scaffold 1301 | 121874 | | 123808 | | reverse | |
| SnBD15_OPHHA | Scaffold 3494 | 129354 | | 133221 | | reverse | |
| SnBD22_OPHHA | Scaffold 3494 | 41134 | | 42372 | | forward | |
| SnBD1_OPHHA | Scaffold 379 | 53328 | | 54822 | | forward | |
| SnBD2_OPHHA | Scaffold 379 | 42936 | | 44192 | | forward | |
| SnBD3_OPHHA | Scaffold 379 | 38315 | | 39679 | | forward | |
| SnBD4_OPHHA | Scaffold 379 | 33224 | | 35101 | | reverse | |
| SnBD5_OPHHA | Scaffold 379 | 22425 | | 24348 | | reverse | |
| SnBD6_OPHHA | Scaffold 379 | 11408 | | 14888 | | reverse | |
| SnBD7_OPHHA | Scaffold 4557 | 59531 | | 61106 | | reverse | |
| SnBD9_OPHHA | Scaffold 4557 | 52381 | | 54904 | | forward | |
| SnBD10_OPHHA | Scaffold 4557 | 41371 | | 55861 | | forward | |
| SnBD13_OPHHA | Scaffold 8908 | 31232 | | 35945 | | forward | |
| SnBD14_OPHHA | Scaffold 9258 | 7431 | | 12206 | | reverse | |
| SnOVOD2_OPHHA | Scaffold 590 | 579737 | | 582510 | | reverse | |
| SnOVOD3_OPHHA | Scaffold 590 | 561523 | | 565826 | | reverse | |
| SnOVOD5_OPHHA | Scaffold 590 | 528352 | | 530354 | | reverse | |
|  |  |  | | |  | | |
| **Species: *Python bivittatus*** | | **Abbreviation: PYTBI** | | | | | |
| **Gene** | **Scaffold** | **Coordinates** | | | | **Strand** | |
| SnBD5_PYTBI | Scaffold 12056 | 2342 | | 2464 | | reverse | |
| SnBD40_PYTBI | Scaffold 1509 | 147901 | | 149380 | | reverse | |
| SNOVOD5_PYTBI | Scaffold2664 | 159417 | | 142159 | | reverse | |
| SnOVOD3_PYTBI | Scaffold3366 | 119769 | | 123364 | | forward | |
| SnOVOD2_PYTBI | Scaffold3366 | 103873 | | 109970 | | reverse | |
| SnOVOD1_PYTBI | Scaffold3366 | 87354 | | 89727 | | reverse | |
| SnBD7_PYTBI | Scaffold 4779 | 79870 | | 80705 | | reverse | |
| SnBD8_PYTBI | Scaffold 4779 | 66501 | | 70429 | | reverse | |
| SnBD9_PYTBI | Scaffold 4779 | 26430 | | 26564 | | forward | |
| SnBD10_PYTBI | Scaffold 4779 | 8719 | | 9116 | | forward | |
| SnBD1_PYTBI | Scaffold 6911 | 10584 | | 11960 | | reverse | |
| SnBD2_PYTBI | Scaffold 6911 | 19146 | | 19749 | | reverse | |
| SnBD3_PYTBI | Scaffold 6911 | 29553 | | 30726 | | reverse | |
| SnBD4_PYTBI | Scaffold 6911 | 38439 | | 40660 | | forward | |
| SnBD22_PYTBI | Scaffold 6958 | 42788 | | 42858 | | forward | |
| SnBD13_PYTBI | Scaffold 7443 | 3565 | | 8594 | | forward | |
| SnBD14_PYTBI | Scaffold 7443 | 22443 | | 25793 | | forward | |
| SnBD15_PYTBI | Scaffold 7672 | 23134 | | 24905 | | reverse | |
| SnBD17_PYTBI | Scaffold 7672 | 5368 | | 7348 | | forward | |
| SnBD6_PYTBI | Scaffold14791 | 601 | | 7224 | | reverse | |
|  | |  | | | | | |
| **Species: *Vipera berus berus*** | | **Abbreviation: VIPBE** | | | | | |
| **Gene** | **Scaffold** | **Coordinates** | | | | | **Strand** |
| SnBD1_VIPBE | Scaffold 2031 | 36708 | | 38649 | | | forward |
| SnBD2_VIPBE | Scaffold 2031 | 30420 | | 31012 | | | forward |
| SnBD3_VIPBE | Scaffold 2031 | 22918 | | 23974 | | | forward |
| SnBD4_VIPBE | Scaffold 2031 | 16302 | | 18215 | | | reverse |
| SnBD5_VIPBE | Scaffold 2031 | 13707 | | 14943 | | | reverse |
| SnBD6_VIPBE | Scaffold 2031 | 6081 | | 6203 | | | reverse |
| SnBD40_VIPBE | Scaffold 3593 | 115514 | | 118331 | | | forward |
| SnBD7_VIPBE | Scaffold 958 | 8341 | | 10878 | | | forward |
| SnBD9_VIPBE | Scaffold 958 | 17060 | | 17191 | | | reverse |
| SnBD10_VIPBE | Scaffold 958 | 33419 | | 35251 | | | reverse |
| SnBD13_VIPBE | Scaffold 958 | 96023 | | 100102 | | | forward |
| SnBD14_VIPBE | Scaffold 958 | 118017 | | 123636 | | | forward |
| SnBD15_VIPBE | Scaffold 958 | 134803 | | 138493 | | | forward |
| SnBD22_VIPBE | Scaffold 958 | 222081 | | 223339 | | | reverse |
| SnBDic2_VIPBE | Scaffold7118 | 2927 | | 13824 | | | forward |
| SnOVOD3_VIPBE | Scaffold 8953 | 2530 | | 6118 | | | forward |
| SnOVOD5_VIPBE | Scaffold 8769 | 27056 | | 29271 | | | forward |
| SnBD39_VIPBE | Scaffold 1562 | 109224 | | 109295 | | | reverse |
|  | |  | | | | | |
| **Species: *Protobothrops mucrosquamatus*** | | **Abbreviation: PROMU** | | | | | |
| **Gene** | **Scaffold** | **Coordinates** | | | | | **Strand** |
| SnBDic1_PROMU | Scaffold 669 | 278063 | 278163 | | | | forward |
| SnBDic2_PROMU | Scaffold 669 | 294284 | 297774 | | | | forward |
| SnOVOD5_PROMU | Scaffold 2965 | 3784 | 6001 | | | | forward |
| SnOVOD3_PROMU | Scaffold 1842 | 90803 | 94288 | | | | forward |
| SnOVOD2_PROMU | Scaffold 1842 | 69700 | 71439 | | | | forward |
| SnOVOD1_PROMU | Scaffold 1842 | 59297 | 61903 | | | | reverse |
| SnBD22_PROMU | Scaffold 173 | 185424 | 186653 | | | | forward |
| SnBD15_PROMU | Scaffold 173 | 283736 | 286300 | | | | reverse |
| SnBD14_PROMU | Scaffold 173 | 294775 | 302266 | | | | reverse |
| SnBD13_PROMU | Scaffold 173 | 326094 | 327620 | | | | reverse |
| SnBD10_PROMU | Scaffold 173 | 406942 | 408507 | | | | forward |
| SnBD9_PROMU | Scaffold 173 | 433331 | 436234 | | | | forward |
| SnBD7_PROMU | Scaffold 173 | 439070 | 440675 | | | | reverse |
| SnBD6_PROMU | Scaffold 173 | 460903 | 465078 | | | | reverse |
| SnBD5_PROMU | Scaffold 173 | 469961 | 470015 | | | | reverse |
| SnBD4_PROMU | Scaffold 173 | 480469 | 482509 | | | | reverse |
| SnBD3_PROMU | Scaffold 173 | 487431 | 488541 | | | | forward |
| SnBD2_PROMU | Scaffold 173 | 493359 | 494015 | | | | forward |
| SnBD1_PROMU | Scaffold 173 | 499084 | 500104 | | | | forward |

Reptile defensin orthologs from *A. carolinensis* [34], *O. hanna* (king cobra) [77], *Python bivittatus* (Burmese python) [78], as well as the pit vipers *V. berus berus* and *P. mucrosquamatus*. Orthologs were identified in published genomes, and genomic locations of ortholog genes are indicated (<https://www.ncbi.nlm.nih.gov/genome/annotation_euk/Protobothrops_mucrosquamatus/100/>) (<https://www.ncbi.nlm.nih.gov/bioproject/170536>) (<https://www.hgsc.bcm.edu/reptiles/european-adder-genome-project>).
